# Association of IL-6 and IL-10 serum levels with genetic variants in diabetic neuropathy: Clinical findings from a Bangladeshi patient cohort

**DOI:** 10.1371/journal.pone.0360933

**Published:** 2026-10-22

**Authors:** Rajib Das, Sheikh Zahir Raihan, Shyamjit Paul, Mohammad Shamsul Arefin Patwary, Abul Kalam Azad Chowdhury

**Affiliations:** 1 Department of Clinical Pharmacy and Pharmacology, Faculty of Pharmacy, University of Dhaka, Dhaka, Bangladesh; 2 Department of Medicine, Dhaka Medical College Hospital, Dhaka, Bangladesh; Noakhali Science and Technology University Faculty of Pharmacy, BANGLADESH

## Abstract

**Background:**

Diabetic Neuropathy (DN) is a prevalent complication of diabetes linked to chronic inflammation. Interleukin-6 (IL-6) and Interleukin-10 (IL-10) are key cytokines implicated in DN pathogenesis. This study investigates the association of IL6 and IL10 gene polymorphisms and serum levels with DN in the Bangladeshi population.

**Materials and methods:**

A case-control study was conducted with 225 participants: diabetic patients with DN (n = 75), diabetic patients without DN (n = 75), and healthy controls (n = 75). Genomic DNA was extracted for genotyping IL-6 (rs1800796) and IL-10 (rs1800896) SNPs via PCR. Serum cytokine levels were measured using ELISA.

**Results:**

Serum IL-6 levels were significantly higher in the DN group than in the other study groups (p < 0.0001), with the highest levels observed among individuals with the IL-6 rs1800796 CC genotype. In unadjusted analyses, the IL-6 CC genotype was associated with increased odds of DN compared with healthy controls (OR = 4.813, 95% CI: 1.752–13.85, p = 0.0040), as was the IL-10 GG genotype (OR = 2.679, 95% CI: 1.000–6.751, p = 0.0373). However, neither IL-6 nor IL-10 genotype remained independently associated with DN after multivariable adjustment. Serum IL-10 levels did not differ significantly among the study groups (p = 0.0948). Combined genotype analyses showed several nominal associations, although these findings were exploratory. Serum IL-6 showed promising discriminatory ability in distinguishing patients with DN from the other study groups, whereas IL-10 demonstrated limited discriminatory performance.

**Conclusion:**

These findings support further investigation of serum IL-6 as a potential candidate biomarker for diabetic neuropathy, whereas the independent contribution of the investigated IL-6 and IL-10 polymorphisms remains uncertain. Larger prospective studies with independent external validation are required before clinical application can be considered.

## Introduction

Diabetes mellitus (DM) is a chronic metabolic disorder characterized by persistent hyperglycemia resulting from impaired insulin secretion, insulin action, or both [1]. Its prevalence is increasing rapidly in Bangladesh because of urbanization, lifestyle changes, and dietary transitions. Approximately 13.1 million adults in Bangladesh were living with diabetes in 2020, and this number is projected to increase substantially by 2045. Diabetic neuropathy (DN) is one of the most common chronic complications of DM and contributes considerably to pain, disability, foot ulceration, hospitalization, and non-traumatic lower-limb amputation [2,3].

DN is a serious complication of DM caused by prolonged high blood sugar levels, leading to nerve damage throughout the body [4]. This condition manifests through several distressing symptoms, the most prevalent of which are pain, which can vary from slight tingling to severe burning or shooting sensations. The symptoms and severity of diabetic neuropathy can vary depending on the type of nerves affected, therefore early identification and treatment are crucial to preventing complications and increasing quality of life [5]. Despite this, the exact mechanisms of DN remain unclear. Pathological changes in DN are linked to oxidative stress, activation of the polyol pathway, accumulation of advanced glycation end products, and activation of protein kinase C, all of which disrupt nerve function [6]. The precise relationship between hyperglycemia and clinical DN is still uncertain. Current hypotheses propose chronic hyperglycemia and related risk factors that may lead to microvascular endothelial damage and neuronal injury through reactive oxygen species and mitochondrial dysfunction. Additionally, research has not clarified the distinctions between painless and painful forms of DN in mechanical and pathological terms [7,8].

Among the key contributors to its pathogenesis are inflammatory processes, in which cytokines such as interleukin-6 (IL-6) and interleukin-10 (IL-10) play pivotal roles [9]. IL-6 is a pro-inflammatory cytokine, while IL-10 functions as an anti-inflammatory mediator, both play critical roles in regulating immune responses and maintaining inflammatory balance [10]. Dysregulation in the expression of these cytokines has been implicated in the development of chronic inflammation, a hallmark of diabetic neuropathy [11]. Variations, or polymorphisms, in the genes encoding IL-6 and IL-10 may lead to altered cytokine expression, influencing susceptibility to neuropathy and affecting its severity in diabetic patients [12]. The SNP rs1800796, referred to as −572 G/C, is a genetic variant located in the promoter region of the IL6 gene on chromosome 7. It influences the expression of IL-6, a crucial cytokine implicated in immunological response, inflammation, and hematopoiesis. On the other hand, the IL-10 rs1800896 polymorphism is a well-studied SNP located in the promoter region of the IL10 gene [13]. This SNP involves guanine (G) to adenine (A) substitution at position −1082, which can affect the transcriptional activity of the IL10 gene, leading to differences in IL-10 production levels [14]. IL-10 is a potent anti-inflammatory cytokine that plays a crucial role in immune regulation by suppressing pro-inflammatory cytokines and promoting immune tolerance. Variations in IL-10 production due to rs1800896 can impact an individual’s immune response and susceptibility to various diseases [14].

The study aimed to explore the association between specific IL6 and IL10 gene polymorphisms along with their serum levels and diabetic neuropathy in the Bangladeshi population. By employing molecular profiling techniques and genetic analysis, this research seeks to identify genetic markers that may predispose individuals to this complication. These findings may improve understanding of the inflammatory mechanisms underlying diabetic neuropathy and provide a basis for future studies investigating inflammatory biomarkers in larger populations.

## Materials and methods

### Study design and participant

Clinical data and blood samples were collected from Dhaka Medical College Hospital, Dhaka, and Chandpur Diabetic Hospital, Chandpur, between June and December 2025. Patients’ medical records were thoroughly reviewed, and relevant demographic and clinical information was documented. Ethical approval for this study was obtained from the Institutional Review Board (IRB) of the Faculty of Pharmacy, University of Dhaka, prior to participant recruitment and sample collection (Approval Ref. No. Fa. Ph. E/052/2025). The study was conducted in accordance with the ethical principles of the Declaration of Helsinki, ensuring the protection of participants’ rights and well-being. Informed written consent was obtained from all participants after providing a clear explanation of the study’s objectives, procedures, potential risks, and benefits. Participant confidentiality was strictly maintained throughout the study, and all collected data were used exclusively for research purposes.

A total of 225 participants were enrolled after applying strict inclusion and exclusion criteria and divided into three groups: Group 1, consisting of Type 2 Diabetes Mellitus (T2DM) patients with diabetic neuropathy (DN) and no other comorbidities (n = 75); Group 2, consisting of T2DM patients without DN or other medical conditions (n = 75); and Group 3, consisting of healthy, non-diabetic individuals with no known chronic diseases (n = 75). Participants were male or female, aged 25–70 years. Pregnant women and individuals with other potential causes of peripheral neuropathy, such as vitamin B12 deficiency, hypothyroidism, chronic alcohol use, chemotherapy history, chronic kidney disease, or other known neurological disorders, were excluded. All participants underwent a standardized neuropathy screening protocol prior to final selection to rule out undiagnosed neurological disorders. Random sampling was applied to select participants for the case-control analysis from each group. The diagnosis of T2DM was based on criteria established by the American Diabetes Association (ADA). Diabetic neuropathy (DN) was defined as peripheral nerve damage, including sensory loss, muscle weakness, pain, and muscle wasting in the lower limbs. DN was assessed through a combination of clinical examination and a validated screening tool, which included 10 g monofilament testing for pressure sensation, vibration perception using a 128 Hz tuning fork, and pinprick sensation evaluation. Additionally, the Michigan Neuropathy Screening Instrument (MNSI) was used to confirm the diagnosis, with a score of ≥2.5 indicating neuropathy. A diagnosis of DN was made based on abnormal clinical findings and/or MNSI criteria, with neuropathy status categorized as a binary variable (present or absent). All evaluations were conducted by trained clinicians adhering to standardized procedures.

### Ethnicity and population characteristics

The study predominantly included participants of Bengali ethnic origin, which represents the majority ethnic group in Bangladesh. While Bangladesh is home to several regional and linguistic subgroups, the recruited participants were largely homogeneous with respect to ethnicity and language. Detailed information on genetic markers or ancestry was not available. However, considering the relatively low genetic differentiation within the Bengali population, the risk of significant population stratification is regarded as minimal, though not entirely excluded.

### Blood sample collection and evaluation of biochemical parameters

From each participant, total 5 mL of venous blood was collected using a 10 mL sterile syringe following standard phlebotomy procedures. From this 5mL of blood, 3 mL was transferred into a BD K2 EDTA VACUTAINER tube for genomic DNA extraction and SNP genotyping. The remaining 2 mL was divided among three separate tubes designated for serum separation and the evaluation of routine biochemical parameters. Blood samples were temporarily stored in an icebox during collection and then transported to the research laboratory, where they were refrigerated at 4°C for 24 hours. After refrigeration, blood samples were fractionated into plasma, buffy coat, and red blood cells (RBCs), which were carefully separated and stored at –20°C for subsequent analyses. Routine biochemical parameters included fasting and random blood glucose (mmol/L), HbA1c (%), total cholesterol, triglycerides, HDL-C and LDL-C (mg/dL), blood urea nitrogen and serum creatinine (mg/dL), C-reactive protein (mg/L), and AST and ALT (U/L). Serum IL-6 and IL-10 concentrations were reported in pg/mL. All biochemical results were interpreted against population-specific reference ranges, and the data were systematically recorded and preserved for further statistical and clinical analyses.

### Genotyping of single nucleotide polymorphisms

Human genomic DNA was extracted from the blood samples using the TIANgen Blood DNA Isolation Kit (China). The quantity and quality of the isolated DNA were evaluated using agarose gel electrophoresis and NanoDrop™ UV-visible spectrophotometry. To examine the relationship between interleukin gene polymorphisms and diabetic neuropathy, two single nucleotide polymorphisms (SNPs) were analyzed: rs1800796 in the *IL6* gene and rs1800896 in the *IL10* gene. Relevant forward and reverse primers were obtained from previous studies (Table 1) [15–17]. For genotyping, the rs1800896 (*IL-10*, −1082 A/G) SNP was analyzed using Amplification Refractory Mutation System PCR (ARMS-PCR), while the rs1800796 (*IL-6*, −572 C/G) SNP was assessed using Tetra-Primer ARMS-PCR (T-ARMS-PCR). PCR reactions were performed in a 25 µL volume containing RNase-free water, master mix, specific primers, and template DNA. Thermal cycling conditions for IL-6 included: initial denaturation at 95°C for 5 minutes; 35 cycles of denaturation at 94°C for 45 seconds, annealing at 64°C for 60 seconds, and extension at 72°C for 120 seconds; followed by a final extension at 72°C for 7 minutes. For IL-10, the conditions were identical except the annealing temperature was set to 67°C. PCR products were analyzed by 2% agarose gel electrophoresis. Amplicons were visualized using a UV transilluminator, and band sizes were determined with a 50 bp DNA ladder. Desired DNA bands were excised with a sterile blade and purified using the TIANgen Gel Extraction Kit, as per manufacturer instructions. Purified products were submitted for Sanger sequencing at Macrogen Inc. (South Korea). Sequencing chromatograms were analyzed using Chromas software, and sequence homology was verified using BLASTn. Genotype confirmation was conducted by aligning the sequences with reference data from the dbSNP database.

**Table 1:** List of Primer Sequence and other properties.

Gene	Primer	Primer Sequence	Bp	GC%	Tm(c)
IL-6 rs1800796 (–572C/G)	Forward Inner	5’- GGCCAGGCAGTTCTACAACAGGCG-3’	24	63	69.3
	Reverse Inner	5’- TGTTCTGGCTCTCCCTGTGTGG-3’	22	59	67.6
	Forward Outer	5’- CTCTAAGTGGGCTGAAGCAGGTGATGA-3’	27	52	66.7
	Reverse Outer	5’- AGTTTCCTCTGACTCCATCGCAGGCC-3’	26	58	69.7
IL-10 rs1800896 (−1082 A/G)	Generic primer	5’-CAGTGCCAACTGAGAATTTGG-3’	21	48	58.9
	Primer G	5’-CTACTAAGGCTTCTTTGGGAG-3’	21	48	57.2
	Primer A	5’-ACTACTAAGGCTTCTTTGGGAA-3’	22	41	57.6

### Measurement of serum IL-6 and IL-10 levels

Quantification of serum IL-6 and IL-10 concentrations was performed using commercially available enzyme-linked immunosorbent assay (ELISA) kits (FineTest, China), following the manufacturer’s protocols. This method provided a reliable and sensitive measurement of inflammatory cytokine levels, offering valuable insights into systemic inflammatory status in diabetic neuropathy.

### Statistical analysis

Statistical analyses were performed using GraphPad Prism version 8.0 (GraphPad Software, San Diego, CA, USA). Continuous variables are presented as mean ± standard error of the mean (SEM), whereas categorical variables are expressed as frequencies and percentages. All statistical tests were two-sided, and a *p*-value < 0.05 was considered statistically significant. Comparisons of continuous variables among the three study groups were performed using one-way analysis of variance (ANOVA). Prior to ANOVA, the assumptions of approximate normality, independence of observations, and homogeneity of variance were assessed, with Levene’s test used to evaluate homogeneity of variance. When the overall ANOVA was statistically significant, Tukey’s multiple-comparison test was applied for post-hoc pairwise comparisons. Prespecified comparisons between two independent groups were performed using the independent-samples *t*-*t*est.

Genotype, allele, and carriage frequencies were compared using Pearson’s chi-square test, whereas Fisher’s exact test was applied when expected cell counts were less than five. Unadjusted odds ratios (ORs) and 95% confidence intervals (CIs) were calculated from 2 × 2 contingency tables. The reference category for each comparison is specified in the corresponding tables. An OR > 1 indicated increased odds, whereas an OR < 1 indicated decreased odds relative to the reference category. To identify factors independently associated with diabetic neuropathy, multivariable binary logistic regression analysis was performed with diabetic neuropathy status (present/absent) as the dependent variable. Based on clinical relevance and significant baseline differences between groups, the model included age, body mass index (BMI), HbA1c, systolic blood pressure, HDL cholesterol, LDL cholesterol, triglycerides, IL-6 rs1800796 genotype, and IL-10 rs1800896 genotype as independent variables. Adjusted odds ratios (AORs) with corresponding 95% CIs were reported. Before model fitting, multicollinearity among independent variables was assessed.

Hardy–Weinberg equilibrium (HWE) was evaluated separately for each single nucleotide polymorphism (SNP) in the healthy control group using Pearson’s chi-square goodness-of-fit test with one degree of freedom. A *p*-value > 0.05 indicated no significant deviation from HWE. Associations between continuous variables were evaluated using Pearson’s correlation coefficient when assumptions of approximate normality, linearity, and absence of influential outliers were satisfied; otherwise, Spearman’s rank correlation coefficient was used.

The discriminatory performance of serum IL-6 and IL-10 concentrations for diabetic neuropathy was explored using receiver operating characteristic (ROC) curve analysis. The area under the curve (AUC) with 95% CIs was calculated, and exploratory optimal cut-off values were determined using the maximum Youden index. Corresponding sensitivity and specificity values were also reported. In the tables, *p* < 0.05 and p < 0.01 indicate nominal statistical significance. As no formal adjustment for multiple comparisons was applied, the results of exploratory genetic and subgroup analyses were interpreted cautiously.

## Results

### Socio-demographic, anthropometric, and biochemical characteristics of participants

The study assessed socio-demographic, anthropometric, and biochemical profiles of three groups: T2DM patients with diabetic neuropathy (DM with DN), T2DM patients without neuropathy (DM without DN), and healthy controls (HC). The DM with DN group had a significantly higher mean age (62.64 ± 0.8455 years) than the DM without DN (54.07 ± 0.9809) and healthy control groups (48.55 ± 1.226) (p < 0.0001), while gender distribution remained balanced across all groups. Urban residency was more prevalent in the DM with DN group (77.33%) compared to the DM without DN (28.00%) and controls (44.00%) (p < 0.0001). Anthropometric measurements showed significantly higher body weight and BMI in the DM with DN group (70.19 ± 1.14 kg, 25.19 ± 0.32 kg/m^2^) compared to other groups (p < 0.0001). The average age of diabetes onset was higher in the DN group (50.83 ± 0.73 years; p = 0.0007), with an average DN duration of 8.33 ± 0.25 years. Both systolic and diastolic blood pressures were significantly elevated in the DM with DN group (p < 0.0001). Biochemically, fasting blood sugar, random blood sugar, and HbA1c levels were significantly higher in the DM with DN group (p < 0.0001). Lipid profiles revealed elevated total cholesterol, triglycerides, and LDL, along with lower HDL in the DN group (p < 0.01). Renal function markers (BUN and creatinine) and inflammatory marker CRP were also significantly increased (p < 0.01), as were liver enzymes AST and ALT (p < 0.01), indicating greater systemic metabolic disturbance in the DM with DN group (Table 2).

**Table 2:** Socio-demographic, anthropometric, and biochemical characteristics of study participants.

Parameters	DM with DN (Mean±SEM)	DM without DN (Mean±SEM)	Healthy Control (Mean±SEM)	p-value
**Age**	62.64 ± 0.8455	54.07 ± 0.9809	48.55 ± 1.226	<0.0001*
**Gender**				
**Male**	38 (50.67%)	36 (48.00%)	37 (49.33%)	ns
**Female**	37 (49.33%)	39 (52.00%)	38 (50.67%)	
**Residence**				
**Urban**	58 (77.33%)	21 (28.00%)	33 (44.00%)	<0.0001**^a,b^
**Rural**	17 (22.67%)	54 (72.00%)	42 (56.00%)	
**Height (cm)**	166.8 ± 0.8425	165.9 ± 0.8336	164.3 ± 0.7052	0.0927
**Weight (kg)**	70.19 ± 1.135	65.36 ± 0.8596	61.88 ± 0.6505	<0.0001*^a,b,c^
**BMI (kg/m**^**2**^)	25.19 ± 0.3211	23.74 ± 0.2344	22.85 ± 0.2021	<0.0001*^a,b,c^
**Age of DM (year)**	50.83 ± 0.7304	47.87 ± 0.8725	–	0.0007*^b^
**Age of DN (year)**	59.16 ± 0.8007	–	–	–
**Time to develop DN from DM (year)**	8.33 ± 0.2523	–	–	–
**Systolic Blood Pressure (mmHg)**	134.9 ± 1.939	121.9 ± 0.7277	116.4 ± 0.7802	<0.0001* ^a,b,c^
**Diastolic Blood Pressure (mmHg)**	93.60 ± 1.817	82.67 ± 0.8768	75.67 ± 0.6599	<0.0001* ^a,b,c^
**Fasting Blood Sugar (mmol/L)**	9.315 ± 0.1986	8.256 ± 0.2246	4.581 ± 0.04319	<0.001*^a,b,c^
**Random Blood Sugar (mmol/L)**	14.81 ± 0.2919	13.42 ± 0.2736	5.876 ± 0.8634	0.0007*^a,b,c^
**Hemoglobin A**_**1c**_ **(%)**	10.41 ± 0.2221	9.153 ± 0.2104	4.745 ± 0.0502	<0.0001*^a,b,c^
**Total Cholesterol (mg/dL)**	208.5 ± 5.564	179.7 ± 3.774	166.1 ± 1.572	0.0071*^a,b^
**Triglycerides (mg/dL)**	240.5 ± 12.46	149.8 ± 6.277	130.3 ± 1.188	0.006*^a,b^
**HDL (mg/dL)**	45.31 ± 1.868	52.56 ± 1.114	48.28 ± 0.4546	0.0004*^b^
**LDL (mg/dL)**	126.6 ± 3.158	104.8 ± 1.719	94.61 ± 0.9514	0.005*^a,b,c^
**BUN (mg/dL)**	21.68 ± 0.7763	17.40 ± 0.7977	12.06 ± 0.4265	0.002*^a,b,c^
**Creatinine (mg/dL)**	1.309 ± 0.0565	1.007 ± 0.0341	0.8133 ± 0.1079	0.0001*^a,b,c^
**C-Reactive Protein (mg/L)**	3.23 ± 0.134	3.16 ± 0.1403	1.89 ± 0.052	0.008*^a,c^
**AST (U/L)**	35.84 ± 1.6	29.13 ± 1.177	26.85 ± 0.6875	<0.001*^a,b^
**ALT (U/L)**	53.41 ± 3.48	38.53 ± 1.743	31.64 ± 0.7778	0.0041*^a,b^

Data are presented as n (%) and mean ± SEM (standard error of the mean) where applicable. The p-values were calculated using the Chi-square test** and ANOVA* as appropriate. All tests were two-sided, and p < 0.05 was considered nominally statistically significant. For ANOVA, Tukey’s multiple-comparison test was used for pairwise comparisons: a; DM with DN vs. Healthy Control, b; DM with DN vs. DM without DN, and c; DM without DN vs. Healthy Control, with significance set at p < 0.05. Abbreviations: ns; not significant.

### Genotypic analysis of IL-6 and IL-10 polymorphisms

The T-ARMS was employed for genotyping the IL-6 rs1800796 [15]. For the G allele, bands of 237 bp and 125 bp are generated, while for the C allele, bands of 237 bp and 157 bp are observed, indicating the presence of the IL-6 rs1800796 (−572 G/C) polymorphism (Fig 1). On the other hand, the ARMS, was employed for genotyping the IL-10 rs1800896 polymorphism, following the method described by Ramesh et al. (2014). In the presence of the A allele at the −1082 position of the IL10 gene, amplification using the A-specific forward primer and the generic reverse primer produces a 259 bp DNA fragment. If the G allele is present at the −1082 position, the G-specific forward primer and the generic reverse primer yield a 258 bp DNA fragment. This strategic primer design ensures accurate identification and differentiation of the IL-10 rs1800896 polymorphism (Fig 2).

**Fig 1:**
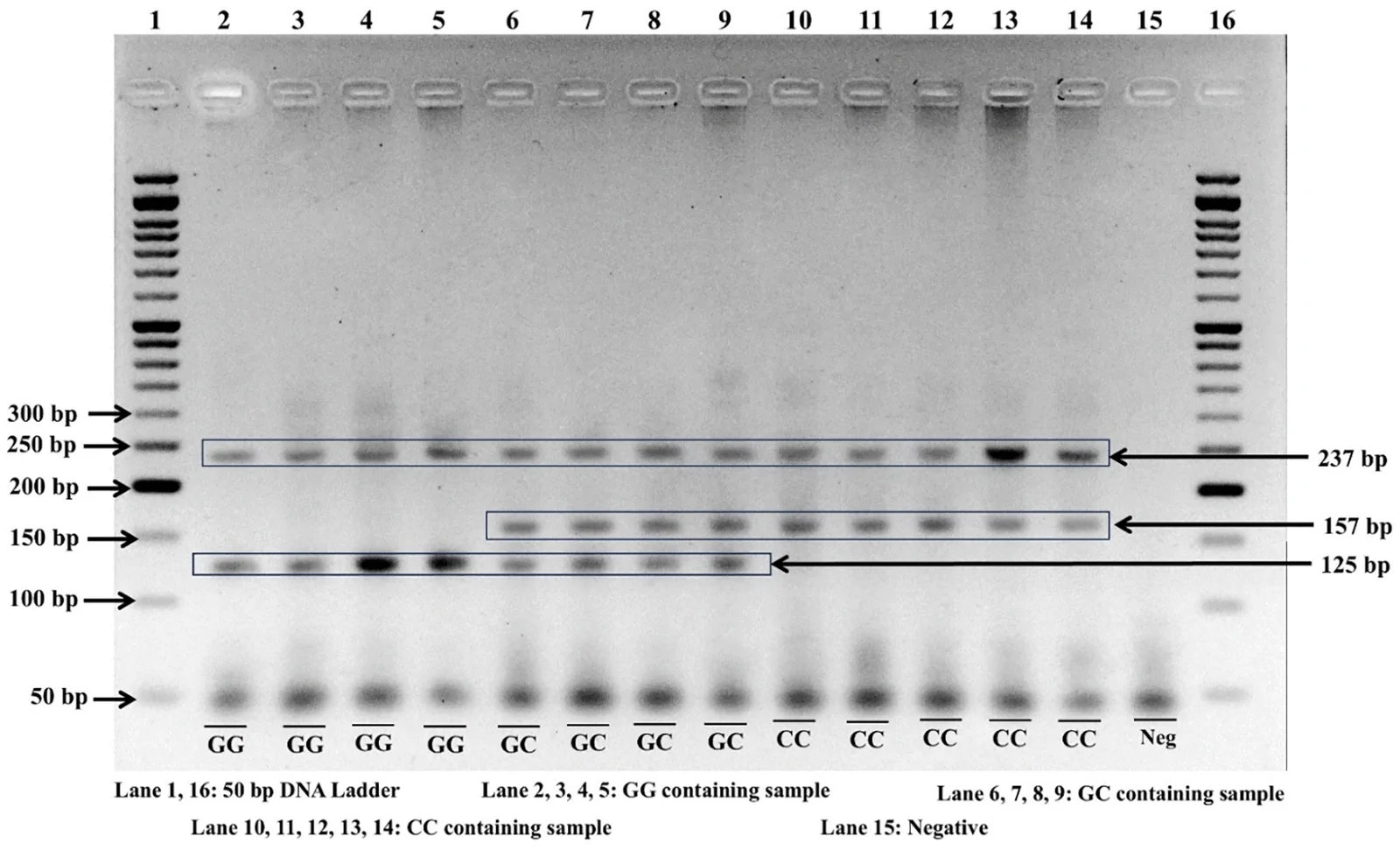
Agarose gel electrophoresis of PCR products for IL-6 rs1800796 genotyping (color-inverted image). The gel image depicts the results of PCR amplification using the Tetra-primer ARMS-PCR technique for genotyping the IL-6 rs1800796 polymorphism. This color-inverted image enhances the contrast for clearer visualization of bands. Lanes 1 and 16 contain the 50 bp DNA ladder for estimating band sizes. Lanes 2–5 show samples with the GG genotype, indicated by bands at 237 bp (control band) and 125 bp. Lanes 6–9 display the GC genotype with bands at 237 bp, 125 bp, and 157 bp. Lanes 10–14 represent the CC genotype, marked by bands at 237 bp and 157 bp. Lane 15 serves as the negative control, exhibiting no bands. The band sizes match the expected PCR product lengths, confirming the successful genotyping of the IL-6 rs1800796 polymorphism. The original uncropped and unadjusted gel image is provided as supplementary file S1 Raw.

**Fig 2:**
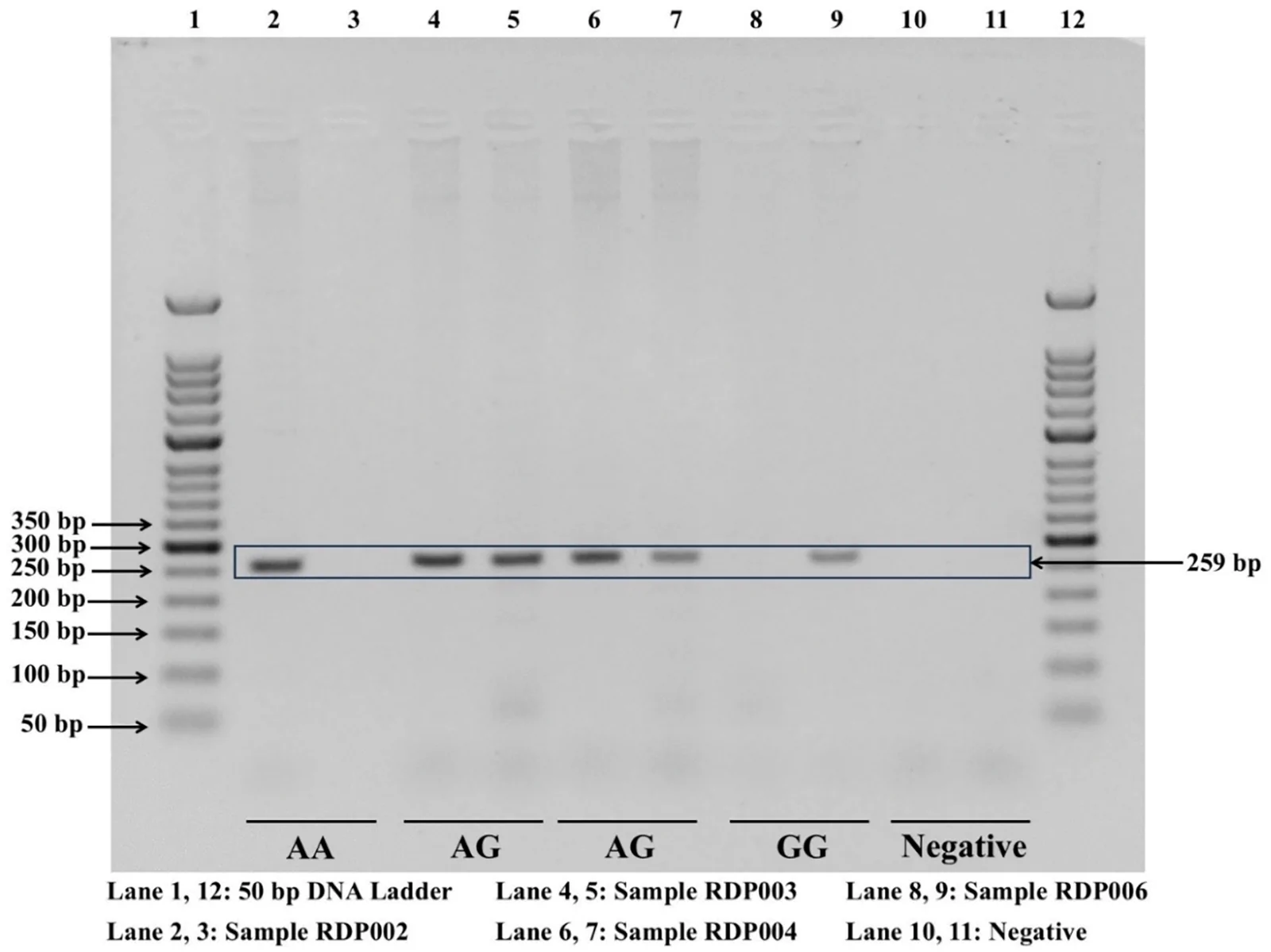
Agarose gel electrophoresis of PCR products for IL-10 rs1800896 genotyping (color-inverted image). The image illustrates PCR products analyzed using the ARMS-PCR method for genotyping the IL-10 rs1800896 polymorphism. This color-inverted image enhances the contrast for better visualization. Lanes 1 and 12 contain the 50 bp DNA ladder for size estimation. Lanes 2 and 3 (AA genotype) show bands at 259 bp. Lanes 4 and 5, and lanes 6 and 7 (AG genotype) show bands confirming heterozygosity at 259 bp. Lanes 8 and 9 (GG genotype) show the expected band at 259 bp, indicating the presence of the G allele. Lanes 10 and 11 are negative controls, showing no amplification. The observed band sizes align with expected results, confirming the successful genotyping of the IL-10 rs1800896 polymorphism. The original uncropped and unadjusted gel image is provided as supplementary file S2 Raw.

The DNA sequencing results confirmed that the amplified PCR fragment corresponded to the IL6 gene, with band sizes of 237 bp and 157 bp, indicating the presence of the C allele at the −572 position. This verification step ensured that the genotyping process for IL-6 was both accurate and reliable, contributing to the overall integrity of the study. For the IL10 gene rs1800896, the polymorphism was positioned 21 bases from the enzyme cut site within the amplicon. Due to technical limitations associated with sequencing immediately adjacent to primer-binding regions, direct confirmation of the IL-10 rs1800896 nucleotide position was not feasible. Despite these constraints, the analysis focused on confirming the IL6 gene and its SNP, as these data were complete and verifiable.

### Genotypic and allelic distributions of IL-6 rs1800796 and IL-10 rs1800896

IL-6 rs1800796 genotypic distribution showed that the GC genotype was most prevalent in both the DM with DN (41.33%) and DM without DN (53.33%) groups, while the GG genotype was most common in the Healthy Control group (50.67%). Allele frequency analysis revealed a decline in the G allele from healthy controls (71.33%) to the DM without DN (54.67%) and DM with DN (54.00%) groups, while the C allele frequency increased progressively from 28.67% in controls to 46.00% in DM with DN. For IL-10 rs1800896, the AG genotype was most common in both the DM with DN (48.00%) and Healthy Control (48.00%) groups, while the AA genotype was more frequent in the DM without DN group (41.33%). The G allele frequency increased from 38.67% in controls to 50.67% in DM with DN, while the A allele frequency decreased from 61.33% in controls to 49.33% in DM with DN. Carriage rates followed similar trends, with the G(+) carriage highest in the DM with DN group (74.67%) (Table 3). The genotype distributions of both investigated polymorphisms were consistent with Hardy–Weinberg equilibrium in the healthy-control group. For IL-6 rs1800796, the observed genotype frequencies (GG = 38, GC = 31, and CC = 6) were comparable to the expected frequencies (GG = 38.16, GC = 30.67, and CC = 6.16), with no significant deviation from HWE (χ^2^ = 0.009, p = 0.924). Similarly, the observed frequencies of IL-10 rs1800896 (AA = 28, AG = 36, and GG = 11) were consistent with the expected frequencies (AA = 28.21, AG = 35.57, and GG = 11.21; χ^2^ = 0.014, p = 0.906). These findings indicate no detectable deviation from HWE in the control group and provide supporting evidence against major genotyping error or substantial population stratification.

**Table 3:** Genotype, allele, and carriage frequencies of IL-6 rs1800796 and IL-10 rs1800896 in healthy controls, DM without DN, and DM with DN groups.

Genotype frequency	Healthy Control		DM without DN		DM with DN	
	Number	Frequency	Number	Frequency	Number	Frequency
IL-6 rs1800796						
GG	38	50.67%	21	28.00%	25	33.33%
GC	31	41.33%	40	53.33%	31	41.33%
CC	6	8.00%	14	18.67%	19	25.33%
IL-10 rs1800896						
AA	28	37.33%	31	41.33%	19	25.33%
AG	36	48.00%	26	34.67%	36	48.00%
GG	11	14.67%	18	24.00%	20	26.67%
Allele frequency						
IL-6 rs1800796						
G	107	71.33%	82	54.67%	81	54.00%
C	43	28.67%	68	45.33%	69	46.00%
IL-10 rs1800896						
A	92	61.33%	88	58.67%	74	49.33%
G	58	38.67%	62	41.33%	76	50.67%
Carriage rate						
IL-6 rs1800796						
G(+)	69	92.00%	61	81.33%	56	74.67%
G(-)	6	8.00%	14	18.67%	19	25.33%
C(+)	37	49.33%	54	72.00%	50	66.67%
C(-)	38	50.67%	21	28.00%	25	33.33%
IL-10 rs1800896						
A(+)	64	85.33%	57	76.00%	55	73.33%
A(-)	11	14.67%	18	24.00%	20	26.67%
G(+)	47	62.67%	44	58.67%	56	74.67%
G(-)	28	37.33%	31	41.33%	19	25.33%

HWE was evaluated in the healthy-control group using Pearson’s chi-square goodness-of-fit test with one degree of freedom. Both SNPs were consistent with HWE (p > 0.05).

### Association of IL-6 rs1800796 and IL-10 rs1800896 genotype distribution with diabetic neuropathy risk

IL-6 rs1800796 genotype analysis showed that the CC genotype was significantly more frequent in patients with DN than in healthy controls (25.33% vs 8.00%; OR = 4.813, 95% CI: 1.752–13.85; p = 0.0040). Similarly, the C allele was more frequent in the DN group than in healthy controls (46.00% vs 28.67%) and was associated with increased odds of DN (OR = 2.120, 95% CI: 1.330–3.458; p = 0.0028). Consistent with the allele-level analysis, C-allele carriage was more frequent in the DN group than in healthy controls (66.67% vs 49.33%) and was associated with increased odds of DN (OR = 2.054, 95% CI: 1.056–3.902; p = 0.0467). Conversely, G-allele carriage was less frequent in the DN group than in healthy controls (74.67% vs 92.00%) and was associated with reduced odds of DN (OR = 0.256, 95% CI: 0.114–0.613; p = 0.0361). No significant allele- or carriage-level difference was observed between patients with and without DN. These unadjusted findings indicate that the IL-6 rs1800796 C allele and CC genotype, as well as the IL-10 rs1800896 G allele and GG genotype, showed nominal associations with DN in selected comparisons with healthy controls (Table 4).

**Table 4:** Comparative analysis of IL-6 rs1800796 genotype and allele frequencies in relation to diabetic neuropathy (DN) risk among study groups.

Genotype	DM with DN vs Healthy Control	DM with DN vs DM without DN	DM without DN vs Healthy Control
**IL-6 rs1800796**			
**CC vs GG**			
**Odds Ratio**	4.813	1.140	4.222
**95% CI**	1.752 - 13.85	0.4412 - 2.713	1.355 - 13.09
**p-value**	0.0040**	0.8214	0.0097**
**GC vs GG**			
**Odds Ratio**	1.520	1.536	2.335
**95% CI**	0.7457 - 3.175	0.7492 - 3.222	1.158 - 4.606
**p-value**	0.2828	0.3436	0.0222*
**CC vs GC**			
**Odds Ratio**	3.167	1.751	1.808
**95% CI**	1.158 - 9.077	0.7849 - 4.133	0.6166 - 5.351
**p-value**	0.0323*	0.2108	0.3129
**IL-10 rs1800896**			
**GG vs AA**			
**Odds Ratio**	2.679	1.813	1.478
**95% CI**	1.000 to 6.751	0.7781 to 4.393	0.5790 to 3.716
**p-value**	0.0373*	0.1982	0.4951
**AG vs AA**			
**Odds Ratio**	1.474	2.259	0.6523
**95% CI**	0.7163 to 3.100	1.083 to 4.976	0.3299 to 1.355
**p-value**	0.3498	0.0387*	0.2770
**GG vs AG**			
**Odds Ratio**	1.818	0.8025	2.266
**95% CI**	0.7795 to 4.269	0.3688 to 1.751	0.8996 to 5.674
**p-value**	0.2008	0.6795	0.1144
**Allele**			
**IL-6 rs1800796**			
**C vs G**			
**Odds Ratio**	2.120	1.027	2.064
**95% CI**	1.330 - 3.458	0.4453 - 1.112	1.293 - 3.367
**p-value**	0.0028**	0.1657	0.0040**
**IL-10 rs1800896**			
**G vs A**			
**Odds Ratio**	1.629	1.458	1.118
**95% CI**	1.027 to 2.539	0.9228 to 2.323	0.6966 to 1.758
**p-value**	0.0482*	0.1319	0.7238
**Carriage rate**			
**IL-6 rs1800796**			
**G(+) vs G(-)**			
**Odds Ratio**	0.256	0.7778	2.641
**95% CI**	0.1631 - 0.8738	0.3809 - 1.553	1.322 - 5.267
**p-value**	0.0361*	0.5955	0.0072**
**C(+) vs C(-)**			
**Odds Ratio**	2.054	0.6764	0.5683
**95% CI**	1.056 - 3.902	0.3185 - 1.478	0.2411 - 1.447
**p-value**	0.0467*	0.4308	0.2612
**IL-10 rs1800896**			
**A(+) vs A(-)**			
**Odds Ratio**	0.4727	0.8684	0.5443
**95% CI**	0.2196 to 1.085	0.4202 to 1.766	0.2486 to 1.236
**p-value**	0.1058	0.8513	0.2144
**G(+) vs G(-)**			
**Odds Ratio**	1.756	2.077	0.8456
**95% CI**	0.8838 to 3.414	1.062 to 4.033	0.4276 to 1.655
**p-value**	0.1587	0.0562	0.7383

Genotype, allele, and carriage frequencies were compared using Pearson’s chi-square test or Fisher’s exact test, as appropriate. Unadjusted ORs and 95% CIs were calculated using the comparison categories indicated in the table. All tests were two-sided. *p < 0.05 and **p < 0.01.

### Analysis of combined effects of IL-6 and IL-10 polymorphisms on diabetic neuropathy risk

The combined genotype analysis of IL-6 rs1800796 and IL-10 rs1800896 revealed that specific genotype pairings were associated with higher odds of diabetic neuropathy. Using the (GG, AG) combination as the reference, individuals with the CC (IL-6) and AA (IL-10) genotype showed a significantly higher risk of DN (OR = 8.00, 95% CI: 1.40–45.76, p = 0.0234), suggesting an eightfold increase in susceptibility. Similarly, the GC/GG combination was also significantly associated with increased risk (OR = 5.33, 95% CI: 1.13–25.12, p = 0.0367). Other combinations, including GG/AA (OR = 1.08) and GC/AA (OR = 1.38), did not show statistically significant associations (p > 0.05), indicating little or no increased risk relative to the reference. A separate analysis using a different reference group also showed a potentially elevated risk for the CC/AA genotype (OR = 4.00, 95% CI: 0.82–19.56, p = 0.0999), although this did not reach statistical significance, likely due to sample size limitations. Most other genotype combinations, such as CC/GG, GG/GG, or GC with various IL-10 genotypes, showed no significant association, with odds ratios near 1 and confidence intervals including 1 (Table 5). These findings suggest that certain IL-6 and IL-10 genotype combinations, particularly CC/AA and GC/GG, may contribute to increased DN susceptibility, though further studies with larger cohorts are needed to confirm these trends. Several combined-genotype categories contained relatively small numbers of participants. Accordingly, the associated odds ratios had wide confidence intervals and should be interpreted as exploratory estimates rather than evidence of definitive gene–gene interactions.

**Table 5:** Combined genotype frequencies of IL-6 rs1800796 and IL-10 rs1800896 and their association with diabetic neuropathy risk compared to healthy controls.

IL-6 (−572 G/C)	IL-10 (−1082 A/G)	DM with DN	Healthy Control	Odds Ratio	95% CI	p-value
CC	GG	5	1	10	1.01-98.88	0.0616
GG	AA	7	13	1.08	0.32-3.64	1
GC	AG	14	12	2.33	0.77-7.09	0.1704
GG	AG	9	18	1	0.32-3.1	1 (Ref)
CC	AA	8	2	8	1.4-45.76	0.0234*
GC	GG	8	3	5.33	1.13-25.12	0.0367*
CC	AG	7	6	2.33	0.6-9.02	0.3049
GC	AA	9	13	1.38	0.43-4.45	0.7665
GG	GG	8	7	2.29	0.63-8.32	0.3256

### Multivariable logistic regression analysis

To determine whether the associations observed in the unadjusted analyses were independent of differences in baseline clinical characteristics, a multivariable binary logistic regression analysis was performed using diabetic neuropathy as the dependent variable. The model was adjusted for age, BMI, HbA1c, systolic blood pressure, HDL cholesterol, LDL cholesterol, triglycerides, and IL-6 rs1800796 and IL-10 rs1800896 genotypes. Among the variables included in the model, HbA1c remained the only independent factor significantly associated with diabetic neuropathy (AOR = 2.91, 95% CI: 1.80–4.71, p < 0.001). Each 1% increase in HbA1c was associated with approximately a 2.9-fold increase in the odds of diabetic neuropathy after adjustment for other clinical variables. In contrast, age, BMI, systolic blood pressure, HDL cholesterol, LDL cholesterol, triglycerides, IL-6 rs1800796 genotype, and IL-10 rs1800896 genotype were not independently associated with diabetic neuropathy after adjustment (all p > 0.05) (Table 6). Compared with the crude analyses, adjustment for established metabolic and cardiovascular risk factors attenuated the associations between IL-6 and IL-10 polymorphisms and diabetic neuropathy, suggesting that part of the observed unadjusted associations may be explained by differences in clinical characteristics between the study groups.

**Table 6:** Multivariable binary logistic regression analysis of factors associated with diabetic neuropathy among patients with type 2 diabetes mellitus.

Variable	Adjusted OR (AOR)	95% CI	p-value
**Age (per year)**	1.00	0.96–1.05	0.840
**BMI (kg/m^2^)**	1.02	0.84–1.24	0.827
**HbA1c (%)**	**2.91**	**1.80–4.71**	**<0.001**
**Systolic blood pressure (mmHg)**	1.01	0.96–1.06	0.630
**HDL cholesterol (mg/dL)**	1.01	0.94–1.09	0.774
**LDL cholesterol (mg/dL)**	1.00	0.96–1.04	0.997
**Triglycerides (mg/dL)**	1.00	0.98–1.02	0.862
**IL-6 rs1800796 (GC vs. CC)**	0.86	0.30–2.49	0.786
**IL-6 rs1800796 (GG vs. CC)**	1.03	0.37–2.89	0.952
**IL-10 rs1800896 (AG vs. AA)**	0.97	0.47–2.01	0.938
**IL-10 rs1800896 (GG vs. AA)**	1.14	0.43–3.04	0.795

Multivariable binary logistic regression was performed using diabetic neuropathy (DM with DN vs. DM without DN) as the dependent variable. The model included age, BMI, HbA1c, systolic blood pressure, HDL cholesterol, LDL cholesterol, triglycerides, IL-6 rs1800796 genotype, and IL-10 rs1800896 genotype. Adjusted odds ratios (AORs) with 95% confidence intervals (CIs) are presented. Statistical significance was defined as p < 0.05.

### Serum levels of IL-6 and IL-10 in diabetic neuropathy, diabetic controls, and healthy controls

Serum IL-6 levels were significantly higher in the DM with DN group (37.24 ± 1.19 pg/mL) compared to both the DM without DN group (19.33 ± 0.59 pg/mL) and healthy controls (14.58 ± 0.60 pg/mL), with all group comparisons showing statistical significance (p < 0.0001). Gender-specific analysis confirmed this trend, with both males and females in the DM with DN group exhibiting the highest IL-6 levels. Genotype-specific analysis of IL-6 rs1800796 revealed that individuals with the CC genotype had the highest IL-6 levels (41.93 ± 2.21 pg/mL) within the DM with DN group, suggesting that individuals with the CC genotype exhibited the highest serum IL-6 concentrations. In contrast, serum IL-10 levels did not show significant differences among the groups (ANOVA p = 0.0948). The DM without DN group had the highest mean IL-10 levels (12.80 ± 1.13 pg/mL), followed by healthy controls (11.23 ± 0.92 pg/mL), and the DM with DN group (10.09 ± 0.69 pg/mL), with no significant gender-specific differences. Genotype analysis of IL-10 rs1800896 also showed no significant associations with IL-10 levels across the study groups. These findings emphasize a strong association between elevated IL-6 levels and diabetic neuropathy, with IL-6 genotypes, particularly the CC genotype, contributing to increased inflammatory responses. Conversely, IL-10 levels appeared less influenced by disease status or genotype, highlighting its limited role in this cohort (Table 7 and Fig 3).

**Table 7:** Serum IL-6 levels among study groups (DM with DN, DM without DN, and healthy control) stratified by gender and IL-6 (−572) genotype.

Cytokine	DM with DN (Mean±SEM) (n = 75)	DM without DN (Mean±SEM) (n = 75)	Healthy Control (Mean±SEM) (n = 75)	p-value
**IL-6**	37.24 ± 1.187	19.33 ± 0.5901	14.58 ± 0.6015	<0.0001^a,b^
**Gender**				
**Male**	38.18 ± 1.843	18.35 ± 0.9445	14.36 ± 0.7385	0.0024 ^a,b^
**Female**	35.88 ± 1.150	20.21 ± 0.6947	15.71 ± 1.475	<0.0001 ^a,b^
**IL-10**	10.09 ± 0.6869	12.80 ± 1.128	11.23 ± 0.9249	0.0948
**Gender**				
**Male**	9.836 ± 1.075	12.69 ± 1.077	15.43 ± 0.870	0.1574
**Female**	10.33 ± 0.8910	12.89 ± 1.703	14.43 ± 0.6957	0.1087
**Genotype** **(IL-6–572)**				
**GG**	34.70 ± 1.831	18.74 ± 1.279	15.70 ± 0.7504	<0.0001 ^a,b^
**GC**	34.52 ± 1.318	19.62 ± 0.7840	13.24 ± 1.696	0.0072 ^a,b,c^
**CC**	41.93 ± 2.213	19.62 ± 1.048	14.82 ± 0.4802	0.0042 ^a,b^
**Genotype** **(IL-10–1082)**				
**AA**	9.817 ± 1.427	12.10 ± 0.6252	9.341 ± 2.838	0.3349
**AG**	9.835 ± 1.049	12.84 ± 2.701	12.27 ± 0.9418	0.5186
**GG**	10.62 ± 1.144	13.36 ± 1.919	11.91 ± 0.6644	0.3624

Data are presented as mean ± SEM (standard error of the mean) where applicable. The p-values were calculated using the ANOVA. A p-value < 0.05 was considered statistically significant when compared to the control group. For ANOVA, Tukey’s multiple comparisons test was used to identify specific group differences, indicated as follows: a; DM with DN vs. Healthy Control, b; DM with DN vs. DM without DN, and c; DM without DN vs. Healthy Control, with significance set at p < 0.05.

**Fig 3:**
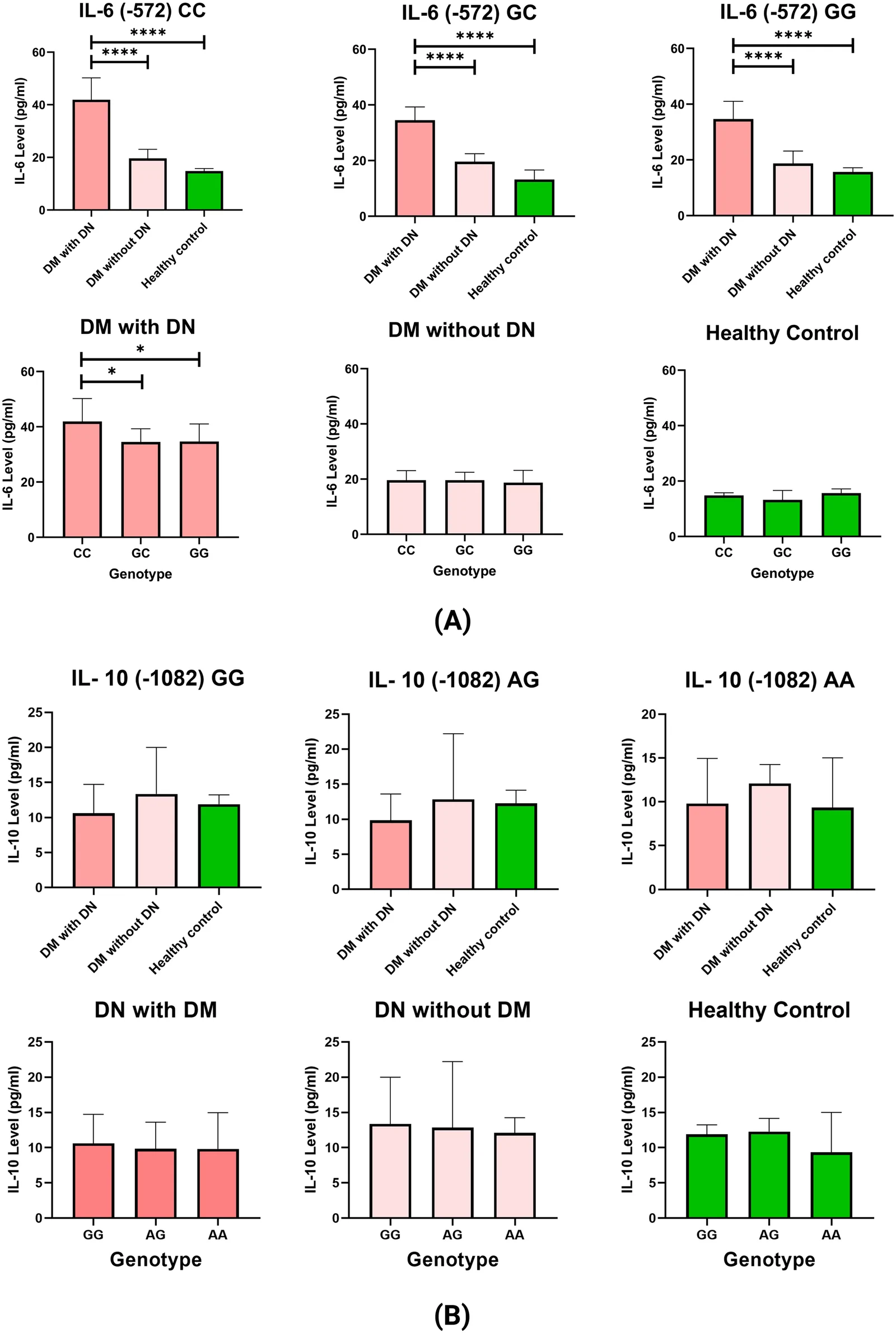
Serum IL-6 and IL-10 levels stratified by genotypes (IL-6 −572 G/C) and IL-10 (−1082) across participant groups. (A) This figure illustrates the serum IL-6 levels (pg/mL) among three genotypic groups (GG, GC, and CC) in participants with DM with DN, DM without DN, and Healthy Controls. (B) The figure represents IL-10 levels in participants with the AA genotype, AG genotype, and GG genotype. No significant differences in IL-10 levels were observed among the three groups for any of the genotypes. Data are expressed as mean ± SEM. Significant differences between groups are denoted by asterisks (**** p < 0.0001).

### Correlation analysis of serum IL-6 and IL-10 levels with clinical parameters

In the DM with DN group, serum IL-6 levels showed a significant positive correlation with both the duration of diabetes (r = 0.4315, p = 0.0061) and the duration of neuropathy (r = 0.3952, p = 0.0128), suggesting that IL-6 levels increase with disease progression (Table 8). However, no significant correlations were observed with age (r = 0.2466, p = 0.1301) or BMI (r = 0.2697, p = 0.0969). In contrast, the DM without DN and healthy control groups showed no significant correlations between IL-6 levels and clinical parameters (p > 0.05). For IL-10, no statistically significant correlations were found across all study groups. In the DM with DN group, IL-10 levels showed weak, non-significant correlations with age (r = 0.0886, p = 0.5918), BMI (r = −0.2749, p = 0.0903), and duration of diabetes (r = 0.1844, p = 0.2611). Similarly, IL-10 levels in the DM without DN and healthy control groups did not correlate significantly with clinical parameters. The findings suggest that IL-6 levels are significantly influenced by disease duration, reflecting its role in chronic inflammation, while IL-10 levels appear to be more stable across disease states.

**Table 8:** Correlation analysis of IL-6 levels with age, BMI, and disease duration in different study groups.

Parameter	Group	IL-6			IL-10		
		r	95% CI	p-value	r	95% CI	p-value
Age	DM with DN	0.2466	−0.0747–0.5216	0.1301	0.08857	−0.233–0.393	0.5918
	DM without DN	0.1234	−0.2138–0.4344	0.4732	−0.2497	−0.538–0.091	0.1480
	Healthy Control	−0.0083	−0.5568–0.5451	0.9784	0.2528	−0.375–0.722	0.4279
BMI	DM with DN	0.2697	−0.0501–0.5393	0.0969	−0.2749	−0.543–0.044	0.0903
	DM without DN	0.08708	−0.2486–0.4041	0.6136	0.01167	−0.322–0.343	0.9469
	Healthy Control	0.2390	−0.3593–0.6980	0.4317	−0.1636	−0.674–0.452	0.6114
Duration of DM	DM with DN	0.4315	0.1342–0.6575	0.0061*	0.1844	−0.139–0.472	0.2611
	DM without DN	0.02675	−0.3045–0.3522	0.8769	−0.3144	−0.586–0.021	0.0658
Duration of Neuropathy	DM with DN	0.3952	0.09105–0.6319	0.0128*	0.2224	−0.100–0.502	0.1736

**r** refers to the correlation coefficient, which measures the strength and direction of the relationship between two variables. Statistically significant differences between groups are indicated by asterisks: *p < 0.05.

### Analysis of IL-6 and IL-10 as potential biomarkers

Receiver operating characteristic curve analysis was performed to explore the ability of serum IL-6 and IL-10 levels to discriminate between the study groups. Serum IL-6 showed promising discriminatory performance in differentiating patients with DN from healthy controls, with an AUC of 0.9034 (95% CI: 0.8280–0.9787; p < 0.0001). At an exploratory cut-off value of 27.26 pg/mL, sensitivity and specificity were 71.79% and 100%, respectively. For differentiating patients with DN from diabetic patients without neuropathy, serum IL-6 produced an AUC of 0.8547 (95% CI: 0.7662–0.9432; p < 0.0001). The corresponding exploratory cut-off value of 30.74 pg/mL yielded a sensitivity of 69.23% and specificity of 100%.

Serum IL-6 showed more modest discriminatory performance in differentiating diabetic patients without neuropathy from healthy controls, with an AUC of 0.6984 (95% CI: 0.5749–0.8218; p < 0.0001). At an exploratory cut-off value of 16.40 pg/mL, sensitivity and specificity were 77.78% and 64.10%, respectively. These findings indicate that serum IL-6 may have potential as a candidate marker for distinguishing patients with DN within this cohort. However, the estimated cut-off values and measures of diagnostic performance should be considered exploratory because they were derived and evaluated in the same relatively small cross-sectional cohort without external validation. In contrast, serum IL-10 showed limited discriminatory performance across all group comparisons, with AUC values below 0.60 (Table 9, Fig 4).

**Table 9:** ROC analysis of IL-6 and IL-10 levels for distinguishing between diabetic neuropathy, diabetic patients without neuropathy, and healthy controls.

Comparison	Cut Off Value (pg/mL)	AUC	95% CI	Sensitivity %	Specificity %	p-value
**IL-6**						
DM with DN vs Healthy Control	27.26	0.9034	0.8280 - 0.9787	71.79	100	<0.0001*
DM with DN vs DM without DN	30.74	0.8547	0.7662 - 0.9432	69.23	100	<0.0001*
DM without DN vs Healthy control	16.40	0.6984	0.5749 - 0.8218	77.78	64.10	0.0031*
**IL-10**						
DM with DN vs Healthy Control	10.87	0.5726	0.4011-0.7442	48.72	75	0.4503
DM with DN vs DM without DN	11.42	0.5982	0.4690 - 0.7274	58.97	62.86	0.1468
DM without DN vs Healthy control	15.20	0.5321	0.3490 - 0.7153	14.29	100	0.7419

Statistically significant differences between groups are indicated by asterisks: *p < 0.05.

**Fig 4:**
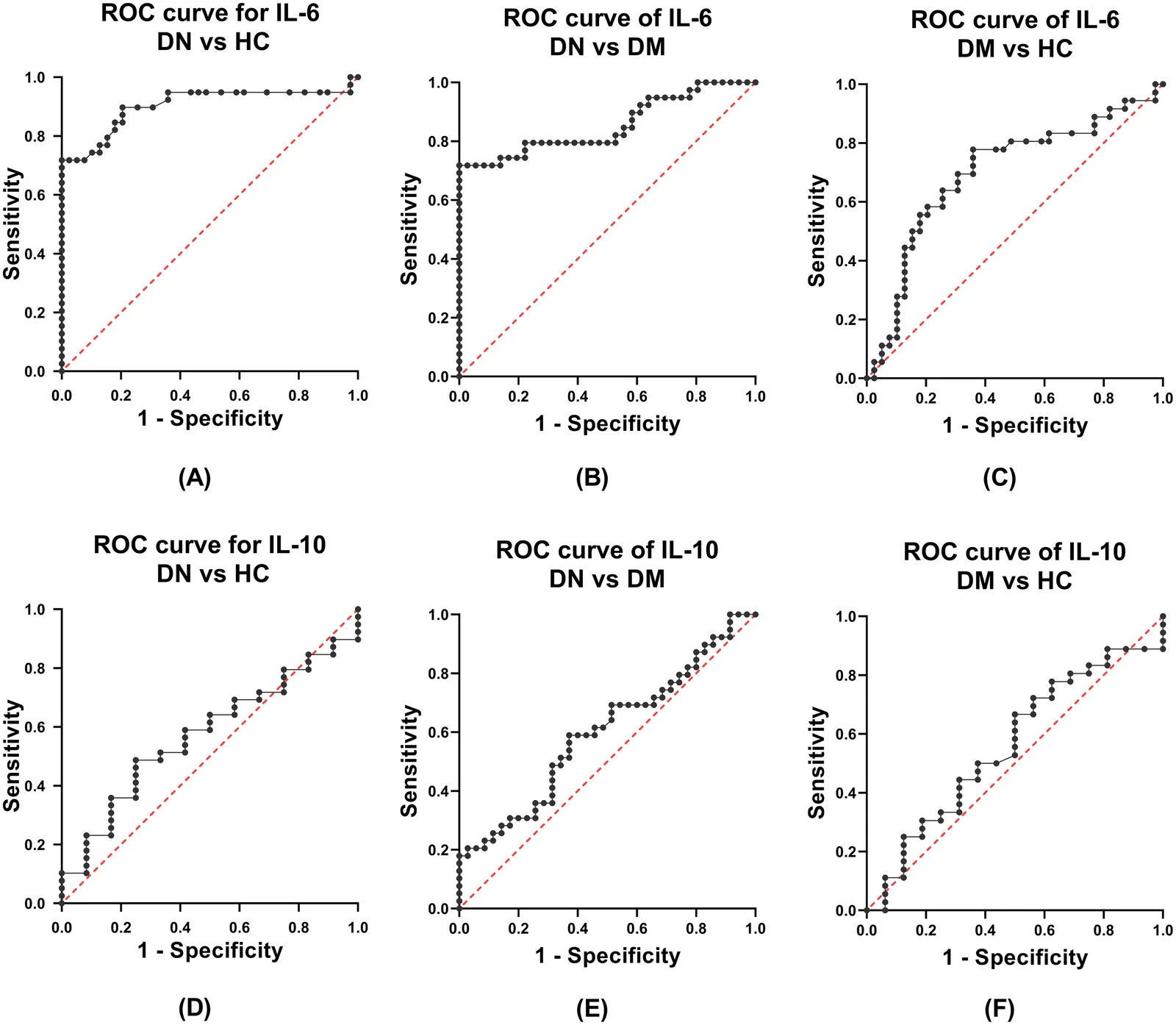
Receiver operating characteristic curves for serum IL-6 and IL-10 levels across the study-group comparisons. The curves illustrate the discriminatory performance of serum IL-6 and IL-10 in differentiating patients with DN, diabetic patients without DN, and healthy controls. The reported AUC values, sensitivity, specificity, and cut-off values are exploratory estimates derived from the present cohort and have not been externally validated. The diagonal line represents discrimination equivalent to chance (AUC = 0.5).

## Discussion

The present study demonstrated that patients with diabetic neuropathy (DN) exhibited distinct demographic, anthropometric, and biochemical characteristics compared with diabetic patients without neuropathy and healthy controls, reinforcing the multifactorial nature of DN. Individuals with DN were older and had a higher body mass index (BMI), elevated blood pressure, and poorer glycaemic control, all of which are well-established risk factors for diabetic microvascular complications. These findings are consistent with previous studies reporting that advancing age, obesity, and hypertension contribute to the development and progression of neuropathy through cumulative metabolic stress, chronic inflammation, endothelial dysfunction, and impaired microvascular perfusion [18–21]. The greater proportion of urban residents among patients with DN may further reflect the influence of lifestyle-related factors, including reduced physical activity, dietary habits, and increasing metabolic risk associated with urbanization [22,23].

The biochemical findings further support the close relationship between metabolic dysregulation and diabetic neuropathy. Patients with DN exhibited significantly higher fasting and random blood glucose levels, together with increased HbA1c concentrations, indicating persistent hyperglycaemia. Chronic hyperglycaemia is a key pathogenic mechanism underlying diabetic neuropathy, promoting oxidative stress, accumulation of advanced glycation end products, activation of inflammatory pathways, and subsequent peripheral nerve injury [24,25] In the present study, multivariable logistic regression further demonstrated that HbA1c remained the only independent predictor of diabetic neuropathy after adjustment for relevant clinical confounders, underscoring the dominant contribution of poor glycaemic control to disease development. The observed crude associations were attenuated after adjustment for clinical covariates, suggesting that metabolic factors, particularly glycemic control, explain a substantial proportion of the genetic association.

In addition to impaired glycaemic control, patients with DN displayed an unfavorable metabolic profile characterized by elevated total cholesterol, triglycerides, LDL cholesterol, inflammatory markers, and renal function indices. These abnormalities are consistent with previous reports linking dyslipidemia, systemic inflammation, and microvascular dysfunction to the progression of diabetic neuropathy [19,26–31]. Elevated AST and ALT levels observed in the DN group may also reflect the metabolic disturbances frequently associated with insulin resistance and chronic diabetes [32]. Collectively, these findings indicate that diabetic neuropathy develops within a complex metabolic milieu involving chronic hyperglycaemia, obesity, hypertension, dyslipidaemia, systemic inflammation, and end-organ dysfunction, highlighting the importance of comprehensive risk-factor management in patients with type 2 diabetes mellitus.

The present study demonstrated that IL-6 rs1800796 showed the most consistent genetic association with diabetic neuropathy among the investigated polymorphisms. The CC genotype, C allele, and C(+) carriage were more frequent in patients with diabetic neuropathy than in healthy controls and were associated with increased odds of disease, whereas G(+) carriage was associated with reduced odds. These findings are consistent with previous studies reporting that the C allele is associated with enhanced IL-6 expression and a heightened inflammatory response, both of which have been implicated in the pathogenesis of diabetic complications [15,33–36]. However, these associations were observed primarily in comparisons with healthy controls and were not statistically significant between diabetic patients with and without neuropathy. Therefore, the observed genetic associations may reflect susceptibility to diabetes-related inflammatory processes rather than neuropathy specifically and should be interpreted cautiously.

Associations involving IL-10 rs1800896 were less consistent. Although the GG genotype and G allele were associated with increased odds of diabetic neuropathy in selected comparisons, these findings were not consistently supported across genotype, allele, and carriage analyses. Moreover, the observed associations were not accompanied by corresponding differences in circulating IL-10 concentrations. Similar inconsistencies have been reported in previous studies, suggesting that the influence of IL-10 polymorphisms may vary across different ethnic populations because of differences in genetic background, environmental exposures, and gene-environment interactions [11,37–39]. Consequently, the contribution of IL-10 rs1800896 to diabetic neuropathy susceptibility remains uncertain and warrants further investigation.

Analysis of combined IL-6/IL-10 genotypes identified several nominally significant associations; however, these findings should be interpreted with caution because many genotype combinations occurred at low frequencies, resulting in wide confidence intervals and imprecise effect estimates. Therefore, the present data do not provide sufficient evidence to support a synergistic interaction between these two cytokine polymorphisms, and larger studies using adequately powered interaction analyses are required to clarify their combined effects.

To determine whether the observed genetic associations were independent of established clinical risk factors, we performed a multivariable logistic regression analysis adjusting for age, BMI, HbA1c, systolic blood pressure, lipid profile, and the investigated cytokine polymorphisms. After adjustment, HbA1c remained the only independent predictor of diabetic neuropathy, whereas the associations of IL-6 rs1800796 and IL-10 rs1800896 were attenuated and were no longer statistically significant. These findings indicate that the crude genetic associations were influenced, at least in part, by differences in metabolic risk factors between the study groups. Nevertheless, patients with diabetic neuropathy consistently exhibited higher circulating IL-6 concentrations, and individuals carrying the IL-6 rs1800796 CC genotype showed the highest serum IL-6 levels, supporting the hypothesis that this polymorphism may contribute to diabetic neuropathy by modulating inflammatory activity rather than acting as an independent clinical predictor. In contrast, the contribution of IL-10 polymorphisms appeared comparatively modest after adjustment. Collectively, these findings support the concept that diabetic neuropathy is a multifactorial disorder in which poor glycaemic control remains the predominant clinical determinant, while IL-6-mediated inflammatory pathways may contribute to disease susceptibility.

The present study further demonstrated that patients with diabetic neuropathy exhibited significantly higher circulating IL-6 concentrations than diabetic patients without neuropathy and healthy controls, supporting the involvement of IL-6-mediated inflammation in the pathogenesis of diabetic neuropathy. Consistent with previous reports, elevated IL-6 levels were associated with neuropathy severity and chronic inflammatory activity [40]. Moreover, individuals carrying the IL-6 rs1800796 CC genotype exhibited the highest serum IL-6 concentrations, suggesting that this polymorphism may influence susceptibility to diabetic neuropathy through modulation of inflammatory responses rather than acting as an independent genetic determinant after adjustment for established clinical risk factors. These findings support IL-6 as a promising candidate biomarker reflecting inflammatory activity in diabetic neuropathy and provide additional evidence for its biological relevance in disease pathogenesis.

In contrast, the findings for IL-10 were considerably less consistent. Serum IL-10 concentrations did not differ significantly among the study groups, nor were they associated with IL-10 rs1800896 genotype or the evaluated clinical characteristics. Although selected genotype and allele comparisons showed nominally significant associations, these findings were not consistently supported across different genetic models and were not accompanied by corresponding changes in circulating IL-10 concentrations. Consequently, the present data do not provide convincing evidence that circulating IL-10 is independently associated with diabetic neuropathy or possesses meaningful discriminatory value in this cohort. The apparent inconsistency between the genetic and biochemical findings may reflect the modest sample size, population-specific genetic effects, limited statistical power, or the influence of additional regulatory mechanisms affecting IL-10 expression that were not evaluated in the present study.

Correlation analyses further supported the potential relevance of IL-6 in disease progression. Serum IL-6 concentrations showed significant positive correlations with both diabetes duration and neuropathy duration, suggesting that prolonged metabolic stress and chronic hyperglycaemia may sustain inflammatory responses during the progression of diabetic neuropathy. These observations are consistent with previous studies linking persistent elevation of IL-6 to chronic inflammation and progressive peripheral nerve injury in long-standing diabetes [41–43]. In contrast, serum IL-10 concentrations showed no significant correlations with demographic or clinical variables, indicating that circulating IL-10 may be less informative as a marker of disease severity or progression in the present population.

Receiver operating characteristic (ROC) analysis demonstrated that serum IL-6 showed promising discriminatory ability for differentiating patients with diabetic neuropathy from diabetic patients without neuropathy and healthy controls within the present cohort. However, these findings should be interpreted cautiously. The observed specificity values may have been overestimated because the optimal cut-off values were derived and evaluated using the same relatively small cross-sectional dataset, and no independent validation cohort was available. Furthermore, the cross-sectional design precludes assessment of the predictive value of IL-6 for future neuropathy development. Therefore, serum IL-6 should be regarded as a potential biomarker or candidate marker, rather than a clinically validated diagnostic biomarker, until its performance is confirmed in larger prospective multicentre studies involving independent populations [44,45]. By comparison, IL-10 demonstrated poor discriminatory performance across all group comparisons, suggesting limited utility as an independent biomarker for diabetic neuropathy.

Several limitations should be considered when interpreting these findings. First, although the study included equal numbers of diabetic patients with neuropathy, diabetic patients without neuropathy, and healthy controls, the overall sample size was relatively modest for genetic association analyses. Consequently, several genotype and combined-genotype categories contained small numbers of participants, resulting in wide confidence intervals and reduced precision of the estimated odds ratios. Second, multiple genotype, allele, carriage, combined-genotype, cytokine, correlation, and ROC analyses were performed without formal adjustment for multiple comparisons. Therefore, some nominally significant findings, particularly those involving infrequent genotype combinations, should be regarded as exploratory and interpreted cautiously. Third, this was a single-centre study conducted in a Bangladeshi population without an independent replication cohort, limiting the generalisability of the findings to other ethnic groups. Finally, although a conventional post-hoc power analysis was not performed because it provides limited information beyond the observed effect estimates and confidence intervals, the wide confidence intervals observed for several subgroup analyses clearly indicate the need for larger, adequately powered multicentre studies with independent external validation to confirm the present findings.

## Conclusion and future directions

This study identified elevated serum IL-6 levels and variation in IL-6 rs1800796 among patients with DN. The exploratory ROC findings suggest that serum IL-6 may be a potential candidate marker for distinguishing patients with DN from diabetic patients without neuropathy and healthy individuals. However, these findings were obtained from a relatively small, cross-sectional cohort, and the proposed cut-off values were not externally validated. Consequently, the clinical diagnostic utility of IL-6 cannot yet be established. Larger prospective, multi-centre studies with independent validation cohorts are necessary before serum IL-6 can be considered for diagnostic or risk-stratification applications. In comparison, the findings related to IL-10 were largely non-significant and inconsistent. Serum IL-10 levels did not differ significantly among the study groups, were not associated with the investigated genotype or clinical variables, and showed limited discriminatory performance. Although a few IL-10 rs1800896 genotype and allele comparisons reached nominal statistical significance, these associations were not consistent across the different analytical models and should be interpreted cautiously. Therefore, the present study does not establish a clear role for IL-10 as a marker or determinant of DN. Larger, prospective studies with independent cohorts, adjustment for relevant confounders, and appropriate correction for multiple comparisons are needed to clarify its potential contribution.

Looking ahead, future research should focus on expanding these findings with larger, longitudinal, and multi-center studies across diverse populations to improve generalizability. The integration of advanced genomic and proteomic technologies will be critical in identifying additional biomarkers and understanding gene-environment interactions. Longitudinal studies will be essential to explore the temporal relationship between cytokine levels, genetic factors, and DN progression, potentially leading to early predictive markers and personalized treatment plans. Future studies should investigate whether integrating cytokine profiles, genetic information, and established clinical risk factors with predictive modeling approaches, including machine learning, can improve the identification of patients at risk of diabetic neuropathy. Such approaches should first be evaluated and externally validated in larger, prospective, multi-centre cohorts before their potential clinical utility can be determined.

## Supporting information

S1 Raw The original uncropped and unadjusted gel image of agarose gel electrophoresis of PCR products for IL-6 rs1800796 genotyping. (TIF)

S2 Raw The original uncropped and unadjusted gel image of agarose gel electrophoresis of PCR products for IL-10 rs1800896 genotyping. (TIF)

S1 File Graphical abstract. (TIF)
