# Supplementary material for: Association of IL-6 and IL-10 serum levels with genetic variants in diabetic neuropathy: Clinical findings from a Bangladeshi patient cohort
Source: PLoS One. 2026 Oct 22;21(10):e0360933. doi: 10.1371/journal.pone.0360933 (PMC13690206; doi:10.1371/journal.pone.0360933)
Supplement: Supplementary file 1 [file pone.0360933.s004.docx]

**Dear Editor and Reviewers,**

We sincerely thank you for the time, effort, and constructive comments provided during the review of our manuscript entitled **"Association of IL-6 and IL-10 Serum Levels with Genetic Variants in Diabetic Neuropathy: Clinical Findings from a Bangladeshi Patient Cohort"** (Manuscript ID: **PONE-D-26-14132**).

We greatly appreciate the insightful suggestions, which have helped us improve the scientific quality, clarity, and overall presentation of our manuscript. We have carefully considered each comment and revised the manuscript accordingly. In the revised version, we have addressed all concerns by performing additional statistical analyses, including multivariable logistic regression and Hardy–Weinberg equilibrium testing, revising the statistical methodology, moderating the interpretation of our findings, improving the language and organization of the manuscript, correcting inconsistencies in the reported data, and updating the Discussion and Conclusion to better reflect the study design and results.

Detailed point-by-point responses to each comment are provided below. For clarity, the reviewers' comments are reproduced in **bold**, followed by our responses. All revisions made in the manuscript have been highlighted using **track changes** (**highlighted text**, as applicable).

We hope that the revised manuscript satisfactorily addresses all of the reviewers' and editor's concerns, and we respectfully submit it for your further consideration.

**Sincerely,**

**Professor Dr Sheikh Zahir Raihan**
*On behalf of all authors*

**Additional Editor Comments:**

The manuscript addresses a clinically relevant topic investigating the association of IL-6 and IL-10 serum levels and genetic polymorphisms with diabetic neuropathy in a Bangladeshi population. The reviewers acknowledged the potential value of the IL-6 findings and the combined biochemical and genetic approach. However, several important methodological, statistical, and interpretative concerns must be addressed before the manuscript can be considered further. Major concerns requiring revision include:

1. The IL-6 allele and carriage analyses appear contradictory in some sections of the manuscript. Please carefully re-evaluate all calculations and ensure that the interpretation of allele frequencies, carriage rates, odds ratios, and associated risk directions are fully consistent throughout the Results and Discussion sections.

**Response:** We sincerely thank the reviewer for identifying this inconsistency. We re-evaluated the IL-6 rs1800796 genotype, allele, and carriage calculations using the frequencies presented in Table 3. This review revealed that the odds ratios for the G(+) and C(+) carriage comparisons had been incorrectly assigned/interpreted in Table 4 and the corresponding Results text. After correction, C(+) carriage was associated with increased odds of DN compared with healthy controls (OR = 2.054), whereas G(+) carriage was associated with reduced odds of DN (OR = 0.256). These corrected findings are consistent with the higher C-allele frequency observed in patients with DN. We also corrected the C-versus-G allele OR for the comparison between patients with and without DN to 1.027, confirming that no significant allele-level difference existed between the two diabetic groups. Table 4 and the corresponding Results and Discussion sections have been revised to ensure consistent presentation of allele frequencies, carriage rates, odds ratios, reference categories, and risk directions. We have also moderated the interpretation because the significant associations were primarily observed against healthy controls rather than between the two diabetic groups.

2. Hardy–Weinberg equilibrium (HWE) analysis for the control group is currently missing. Please perform and report HWE testing for both investigated SNPs to support genotyping reliability and population validity.

**Response:** We thank the reviewer for this important suggestion. Hardy–Weinberg equilibrium was evaluated for both investigated SNPs in the healthy-control group using Pearson’s chi-square goodness-of-fit test. Neither IL-6 rs1800796 (χ² = 0.009, p = 0.924) nor IL-10 rs1800896 (χ² = 0.014, p = 0.906) showed a significant deviation from HWE. The Statistical Analysis and Results sections have been updated accordingly, and the HWE results have also been incorporated into Table 3. These findings provide supporting evidence against major genotyping errors or substantial population stratification in the control group.

3. The diabetic neuropathy group differs substantially from the diabetic non-neuropathy group in several important clinical variables, including age, BMI, HbA1c, blood pressure, and lipid profile. Since these factors are known contributors to diabetic neuropathy and inflammation, multivariate logistic regression analysis adjusting for key confounders should be performed. Adjusted odds ratios with corresponding 95% confidence intervals should be reported.

**Response:** We sincerely thank the reviewer for this valuable suggestion. Following this recommendation, we performed a multivariable binary logistic regression analysis using diabetic neuropathy status as the dependent variable. The model was adjusted for clinically relevant confounding variables, including age, sex, BMI, HbA1c, systolic blood pressure, HDL cholesterol, LDL cholesterol, triglycerides, and IL-6 and IL-10 genotypes. Adjusted odds ratios (AORs) with corresponding 95% confidence intervals have been added to the revised manuscript. After adjustment, HbA1c remained an independent predictor of diabetic neuropathy, whereas the associations of IL-6 and IL-10 polymorphisms were attenuated and were no longer statistically significant. The Statistical Analysis and Results sections have been revised accordingly, and a new table summarizing the multivariable logistic regression findings has been included in the revised manuscript.

4. The manuscript currently overstates the diagnostic significance of IL-6. ROC findings should be interpreted more cautiously. An excellent AUC does not indicate “perfect” diagnostic performance, particularly in a relatively small cross-sectional cohort without external validation. Please revise the wording throughout the manuscript and replace definitive terms such as “diagnostic biomarker” with more cautious terminology such as “potential biomarker” or “candidate marker.”

**Response:** We thank the reviewer for this important observation. We agree that the original manuscript overstated the diagnostic implications of the ROC findings. We have revised the Abstract, Results, Discussion, Conclusions, and Figure 4 caption to describe serum IL-6 as a “potential biomarker” or “candidate marker” and its ROC performance as “promising discriminatory ability within the present cohort.” Definitive expressions such as “diagnostic biomarker,” “perfect diagnostic performance,” “excellent diagnostic accuracy,” and “actionable cut-off values” have been removed or modified throughout the manuscript. We have also clarified that the reported cut-off values are exploratory estimates derived and evaluated in the same relatively small cross-sectional cohort. The revised manuscript now explicitly states that larger prospective, multicentre studies with independent external validation are required before the diagnostic or clinical utility of serum IL-6 can be established.

5. The interpretation of IL-10 findings should be moderated. Since most IL-10-related analyses were statistically non-significant, the Discussion and Conclusion sections should better reflect these limitations and avoid overinterpretation.

**Response:** We thank the reviewer for this important observation. We agree that the original interpretation of the IL-10 findings was stronger than warranted by the overall results. We have therefore revised the Abstract, Discussion, and Conclusions to emphasize that serum IL-10 concentrations did not differ significantly among the study groups, were not significantly associated with the investigated genotype or clinical parameters, and showed limited discriminatory performance. We have also clarified that only selected IL-10 rs1800896 genotype and allele comparisons reached nominal statistical significance and that these associations were not consistent across the different genetic models or supported by corresponding changes in serum IL-10 levels. Accordingly, we now describe the IL-10 findings as preliminary and inconclusive and avoid definitive statements regarding its role in DN. The revised manuscript further acknowledges the modest sample size, potential effects of multiple comparisons, and need for independent validation in larger, adequately powered cohorts.

6. The discrepancy in sample numbers presented in Table 6 requires clarification. Please explain why reduced sample sizes were used in the cytokine subgroup analyses.

**Response:** We sincerely thank the reviewer for identifying this discrepancy. The reduced sample sizes reported in Table 6 resulted from an inadvertent typographical error and did not represent a cytokine subgroup analysis. Serum IL-6 and IL-10 measurements were performed for all participants included in the three study groups (DM with DN, n = 75; DM without DN, n = 75; and healthy controls, n = 75). We have corrected the sample numbers in Table 6 and carefully reviewed the corresponding Results and Methods sections to ensure consistency throughout the revised manuscript. We apologize for the oversight.

7. The relatively small sample size and low frequencies of several genotype combinations raise concerns regarding statistical stability. Some subgroup analyses produced wide confidence intervals and potentially unstable odds ratios. Please discuss these limitations more thoroughly and consider including a post-hoc statistical power analysis.

**Response:** We thank the reviewer for highlighting this important limitation. We have expanded the Limitations and Discussion sections to clarify that the modest sample size and low frequencies of several individual and combined genotypes resulted in sparse cells, wide confidence intervals, and potentially unstable odds-ratio estimates. We now describe these subgroup findings as exploratory and hypothesis-generating and have removed wording implying definitive gene–gene synergy. We have also emphasized the potential influence of multiple testing and the need for validation in larger, independently recruited cohorts. We carefully considered conducting an observed post-hoc power analysis. However, because observed post-hoc power is mathematically dependent on the observed effect size and p-value, it provides little additional information beyond the reported 95% confidence intervals and may produce misleading reassurance for significant findings. We therefore assessed statistical uncertainty primarily through the width of the confidence intervals and the underlying genotype frequencies. The wide intervals observed for several subgroup comparisons clearly demonstrate limited precision. This rationale has been added to the revised manuscript, together with a recommendation that future studies use prospective sample-size calculations based on prespecified clinically meaningful effect sizes.

8. Please verify and standardize all biochemical units throughout the manuscript, including CRP measurements.

**Response:** We thank the reviewer for identifying this issue. We carefully reviewed and standardized the units of all biochemical and cytokine measurements throughout the Methods, Results, tables, and figure captions. Fasting and random blood glucose are now consistently reported in mmol/L; lipid parameters, blood urea nitrogen, and serum creatinine in mg/dL; AST and ALT in U/L; and serum IL-6 and IL-10 in pg/mL. CRP values were converted from mg/dL to mg/L using the conversion factor 1 mg/dL = 10 mg/L. Accordingly, the CRP values for the DM with DN, DM without DN, and healthy-control groups are now reported as 3.23 ± 0.134, 3.16 ± 1.403, and 1.89 ± 0.052 mg/L, respectively. Unit capitalization and formatting including pg/mL, mg/dL, mmol/L, and U/L, have also been standardized throughout the revised manuscript.

9. The manuscript requires substantial language revision. Numerous grammatical, typographical, and sentence-structure issues are present throughout the text. In addition, the Introduction and Discussion sections contain repetitive statements that should be reduced to improve clarity and conciseness.

**Response:** We thank the reviewer for this important observation. The manuscript has undergone comprehensive language revision to correct grammatical, typographical, punctuation, and sentence-structure errors. Terminology, abbreviations, tense, capitalization, and statistical expressions have also been standardized throughout the manuscript. In addition, the Introduction was substantially condensed to focus on the clinical relevance of diabetic neuropathy, its inflammatory basis, the investigated polymorphisms, the existing knowledge gap, and the study objectives. The Discussion was reorganized and shortened to reduce repetition of the Results, consolidate overlapping interpretations of IL-6 and IL-10 findings, and provide a more concise and cautious discussion of the genetic and ROC analyses. Repetitive and speculative statements concerning diagnostic application, personalized treatment, and future research were removed or moderated. These revisions have improved the clarity, readability, and scientific precision of the manuscript.

10. Please ensure that all statistical methods are described clearly and consistently, including the specific post-hoc tests, assumptions used for regression analyses, and criteria applied for statistical significance.

**Response:** We thank the reviewer for this valuable suggestion. The Statistical Analysis section has been thoroughly revised to provide a clearer and more comprehensive description of all statistical procedures used in this study. Specifically, we have:

- identified Tukey's multiple-comparison test as the post-hoc procedure following one-way ANOVA;
- described the assumptions assessed before applying parametric analyses, including approximate normality, independence of observations, and homogeneity of variance (Levene's test);
- expanded the description of the multivariable binary logistic regression analysis by specifying the dependent variable, the covariates included in the model, the reporting of adjusted odds ratios (AORs) with 95% confidence intervals, and the model fit assessment;
- clarified the statistical methods used for genotype analysis, Hardy–Weinberg equilibrium testing, correlation analyses, and ROC curve analysis; and
- consistently stated that all statistical tests were two-sided and that p < 0.05 was considered statistically significant throughout the manuscript.

These revisions improve the transparency and reproducibility of the statistical methodology and fully address the reviewer's concern.

11. The conclusions should remain appropriately cautious and consistent with the cross-sectional design of the study. Statements implying causality or clinical applicability beyond the presented data should be avoided

**Response:** We thank the reviewer for this important comment. The Conclusion has been revised to ensure that the interpretation of our findings is consistent with the observational study design. Statements implying causality or immediate clinical applicability have been moderated. Specifically, we now describe IL-6 as a promising candidate biomarker rather than a confirmed diagnostic marker and explicitly state that the observational nature of the study does not permit causal inference. We further emphasize that larger prospective, multicentre studies are required to validate these findings before any clinical application can be considered.

**Reviewer 1:**

1. The IL-6 allele and carriage analyses are contradictory, so please re-evaluate the calculations and ensure consistent interpretation across all analyses.

**Response:** We sincerely thank the reviewer for identifying this inconsistency. We carefully re-evaluated the genotype, allele, and carriage frequency analyses for IL-6 rs1800796, recalculated the odds ratios and corresponding 95% confidence intervals, and verified all calculations. The carriage analyses have been corrected to ensure consistency with the genotype and allele analyses, and the corresponding interpretations in the Results section have been revised accordingly. In addition, we clarified the reference categories used for odds ratio calculations in the Statistical Analysis section to improve transparency and consistency. These revisions ensure that the genotype, allele, and carriage analyses are internally consistent throughout the manuscript.

2. The ROC results are misinterpreted as “perfect,” so please revise the description to accurately reflect that the AUC indicates excellent, not perfect, diagnostic performance.

**Response:** We thank the reviewer for this important observation. We have revised the interpretation of the ROC analysis throughout the manuscript to avoid overstating the diagnostic performance of IL-6. The term **"perfect"** has been removed and replaced with more appropriate terminology, such as **"excellent"**, **"strong"**, or **"promising diagnostic performance"**, consistent with the observed AUC values. We also emphasize that these findings should be interpreted cautiously and require validation in larger, prospective, multicentre cohorts before any clinical application. These revisions ensure that the interpretation of the ROC analysis is accurate and consistent with accepted reporting standards.

3. Hardy–Weinberg equilibrium testing is missing, so please perform and report HWE analysis for the control group.

**Response:** We thank the reviewer for this valuable suggestion. In the revised manuscript, we performed Hardy–Weinberg equilibrium (HWE) analysis for both IL-6 rs1800796 and IL-10 rs1800896 using genotype data from the healthy control group. The HWE methodology has been described in the Statistical Analysis section, and the results have been incorporated into the Results section. Both polymorphisms were found to be in Hardy–Weinberg equilibrium in the healthy control group (IL-6 rs1800796: χ² = 0.009, p = 0.924; IL-10 rs1800896: χ² = 0.014, p = 0.906), indicating no significant deviation from equilibrium and supporting the reliability of the genotyping data. These revisions address the reviewer's concern.

4. The interpretation of IL-10 findings is overstated, so please revise the discussion to reflect the largely non-significant results.

**Response:** We thank the reviewer for this valuable comment. We have revised the Discussion and Conclusion to provide a more balanced interpretation of the IL-10 findings. The revised manuscript now clearly states that serum IL-10 levels did not differ significantly among the study groups and that the observed associations involving IL-10 rs1800896 were limited and should be interpreted with caution. We have avoided overstating the biological or clinical significance of IL-10 and now emphasize that its role in diabetic neuropathy remains uncertain. We also acknowledge that larger, prospective studies are required to clarify the potential contribution of IL-10 polymorphisms and circulating IL-10 levels to diabetic neuropathy susceptibility. These revisions ensure that our interpretation accurately reflects the predominantly non-significant findings and is fully supported by the presented data.

5. The discrepancy in sample sizes (Table 6) is unclear, so please explain the reason for the reduced sample numbers.

**Response:** We thank the reviewer for carefully identifying this inconsistency. Upon re-examination of the data, we found that the discrepancy in the sample sizes reported in **Table 6** resulted from a typographical error in the original manuscript rather than from any exclusion of participants or missing data. The table has been corrected in the revised manuscript, and the reported sample sizes are now consistent with the study population and all other analyses. We appreciate the reviewer for bringing this issue to our attention, which has helped improve the accuracy and clarity of the manuscript.

6. Confounding variables were not adjusted for, so please perform multivariate analysis (logistic regression) including key covariates.

**Response:** We sincerely thank the reviewer for this valuable suggestion. In response, we performed a multivariable binary logistic regression analysis to evaluate whether the associations with diabetic neuropathy remained after adjustment for important clinical confounders. The model included clinically relevant covariates, namely age, sex, body mass index (BMI), HbA1c, systolic blood pressure, lipid profile, and IL-6 rs1800796 and IL-10 rs1800896 genotypes. Adjusted odds ratios (AORs) with corresponding 95% confidence intervals (CIs) have been calculated and reported in a new table. The Statistical Analysis, Results, and Discussion sections have been revised accordingly to describe the methodology and interpret the adjusted findings. These revisions address the reviewer's concern by accounting for potential confounding factors and providing adjusted estimates of association.

7. There are grammatical and typographical errors, so please perform thorough language editing.

**Response:** We thank the reviewer for this helpful suggestion. The manuscript has been thoroughly revised to improve the quality of the English language. Grammatical, typographical, punctuation, and sentence-structure errors have been corrected throughout the manuscript. We also revised several sections to improve clarity, readability, and scientific writing style while reducing repetitive statements and ensuring consistent terminology. We believe these revisions have substantially improved the overall presentation and readability of the manuscript.

8. The Introduction and Discussion are somewhat repetitive, so please reduce redundancy and improve conciseness.

**Response:** We thank the reviewer for this constructive suggestion. We carefully revised both the Introduction and Discussion to improve conciseness and eliminate repetitive content. Redundant descriptions of diabetes, diabetic neuropathy, inflammatory mechanisms, and cytokine biology were removed or consolidated, while preserving the essential scientific background and interpretation of the findings. The Discussion was also reorganized to improve the logical flow and avoid repetition of previously presented results. These revisions have enhanced the clarity, readability, and overall coherence of the manuscript.

9. Some biochemical units (CRP) may be inconsistent, so please verify and standardize all units.

**Response: We** thank the reviewer for identifying this issue. We carefully reviewed all biochemical parameters throughout the manuscript and verified that the reported units are now consistent and correctly presented. In particular, the units for C-reactive protein (CRP) were corrected and standardized, and all biochemical measurements, including glucose, HbA1c, lipid profile, renal function markers, liver enzymes, and cytokine concentrations, were checked for consistency across the text and tables. These revisions improve the accuracy and consistency of data presentation throughout the manuscript.

**Reviewer 2**

The manuscript by Das et al. addresses a clinically relevant topic on the relationship between inflammatory cytokines (IL-6 and IL-10), their genetic polymorphisms, and diabetic neuropathy (DN) in a Bangladeshi cohort. The study combines biochemical and genetic analyses and the findings regarding IL-6 are potentially valuable that may contribute to understanding inflammatory mechanisms underlying DN. However, the manuscript has several important methodological, statistical, and interpretative weaknesses that must be addressed before the work can be considered for publication.

1. The DN group differs substantially from the diabetic non-DN group in age, BMI, HbA1c, blood pressure, and lipid profile. These are all known risk factors for diabetic neuropathy and inflammatory activation. The current analysis relies mainly on univariate comparisons and crude odds ratios. Without multivariate logistic regression adjusting for these confounders, it is difficult to determine whether the observed associations between IL-6/IL-10 polymorphisms and DN are independent. The authors should perform multivariate logistic regression analysis for DN risk and adjusted ORs with 95% CIs should be reported.

**Response:** We sincerely thank the reviewer for this valuable and constructive suggestion. In response, we performed a multivariable binary logistic regression analysis to evaluate whether the associations between IL-6 rs1800796 and IL-10 rs1800896 polymorphisms and diabetic neuropathy remained after adjustment for important clinical confounders. The regression model included age, sex, body mass index (BMI), HbA1c, systolic blood pressure, lipid profile, and IL-6 and IL-10 genotypes as independent variables, while diabetic neuropathy status (present/absent) was used as the dependent variable. Adjusted odds ratios (AORs) with corresponding 95% confidence intervals (95% CIs) have been calculated and are presented in a newly added table.

In addition, the Statistical Analysis section has been revised to describe the multivariable logistic regression methodology, including the variables included in the model and the reporting of adjusted estimates. The Results section has been updated to present the adjusted findings, and the Discussion has been revised to interpret these results in the context of potential confounding. Following adjustment, the observed associations between IL-6 and IL-10 polymorphisms and diabetic neuropathy were attenuated, indicating that part of the crude associations was explained by differences in important clinical characteristics between the study groups. These revisions address the reviewer's concern and provide a more robust assessment of the independent associations between inflammatory cytokine polymorphisms and diabetic neuropathy.

2. Genetic association studies must evaluate Hardy-Weinberg equilibrium (HWE) in controls to assess genotyping reliability and population validity. The manuscript does not report HWE testing for either SNP.

**Response:** We sincerely thank the reviewer for this important suggestion. In the revised manuscript, we performed Hardy–Weinberg equilibrium (HWE) analysis for both IL-6 rs1800796 and IL-10 rs1800896 using genotype data from the healthy control group. The HWE methodology has been incorporated into the Statistical Analysis section, and the results have been reported in the Results section. Both polymorphisms were found to be consistent with Hardy–Weinberg equilibrium (IL-6 rs1800796: χ² = 0.009, *p* = 0.924; IL-10 rs1800896: χ² = 0.014, *p* = 0.906), indicating no significant deviation from equilibrium. These findings support the quality and reliability of the genotyping data and reduce concerns regarding genotyping error or substantial population stratification. We believe these revisions adequately address the reviewer's concern.

3. The study includes only 75 subjects per group. For genetic association studies involving multiple genotype comparisons and combined genotype analyses, this sample size is relatively small. Several subgroup analyses contain low combined genotype frequency count (e.g., CC/GG), resulting in very wide confidence intervals, unstable OR estimates, and risk of false-positive findings. The authors should include more participants and/ or include post-hoc power calculation.

**Response:** We thank the reviewer for this important observation. We acknowledge that the sample size of our study is relatively modest for genetic association analyses, particularly for subgroup and combined genotype comparisons. As the study has already been completed, it was not feasible to recruit additional participants during the revision process. To address this concern, we have revised the Discussion to explicitly acknowledge that the limited sample size may have resulted in wider confidence intervals and reduced precision for some subgroup estimates. Accordingly, we have emphasized that these findings should be interpreted cautiously and considered exploratory until confirmed in larger, independent studies. Although post-hoc (observed) power analysis has been proposed in some studies, it is generally not recommended because it provides limited additional information beyond the observed effect estimates and confidence intervals. Instead, we report effect sizes with 95% confidence intervals, allowing readers to assess the precision of the estimates directly. We have therefore highlighted the need for larger, multicentre studies with greater statistical power to validate the observed associations.

4. The manuscript repeatedly describes IL-6 as a “diagnostic biomarker” with strong clinical applicability. However, the study is cross-sectional, no validation cohort was used, and specificity values of 100% are suspicious in small cohorts. Therefore, conclusions regarding diagnostic utility are premature. The authors should moderate claims and use language such as potential biomarker or candidate marker.

**Response:** We sincerely thank the reviewer for this thoughtful and important comment. We agree that the observational nature of our study and the absence of an independent validation cohort preclude definitive conclusions regarding the diagnostic utility of IL-6. Accordingly, we have carefully revised the manuscript to moderate our interpretation throughout the Abstract, Discussion, and Conclusion. Specifically, we replaced expressions such as "diagnostic biomarker" with more appropriate terms, including "potential biomarker" and "promising candidate biomarker", and we have avoided statements implying immediate clinical applicability. In addition, we now explicitly acknowledge that the reported diagnostic performance should be interpreted cautiously because it was derived from a relatively small cohort without external validation. We emphasize that larger prospective, multicentre studies with independent validation cohorts are required before IL-6 can be considered for clinical application. These revisions ensure that the conclusions are fully consistent with the study design and the strength of the presented evidence.

5. The manuscript contains numerous grammatical errors, such as
“both play critical role” → “both play critical roles”
“This condition appears in a number of distressing symptoms” → “This condition manifests with several distressing symptoms”
“such DN being prevalent” → “with DN being prevalent”

**Response:** We sincerely thank the reviewer for carefully identifying these language issues. The manuscript has been thoroughly revised to improve grammar, sentence structure, punctuation, and overall readability. The specific examples highlighted by the reviewer have been corrected, and the entire manuscript has undergone comprehensive language editing to ensure consistent scientific writing and improve clarity throughout. We believe these revisions have substantially enhanced the overall quality and presentation of the manuscript.

Once again, we sincerely thank the Editor and the Reviewers for their careful evaluation and constructive comments. We believe that the revisions made in response to these suggestions have substantially improved the scientific rigor, clarity, and overall quality of the manuscript. We hope that the revised manuscript is now suitable for publication in PLOS ONE.
