# Supplementary material for: Association of IL-6 and IL-10 serum levels with genetic variants in diabetic neuropathy: Clinical findings from a Bangladeshi patient cohort
Source: PLoS One. 2026 Oct 22;21(10):e0360933. doi: 10.1371/journal.pone.0360933 (PMC13690206; doi:10.1371/journal.pone.0360933)
Supplement: Supplementary file 2 [file pone.0360933.s005.docx]

Dear Editor,

We sincerely thank you and the reviewers for the careful evaluation of our manuscript and for providing constructive comments and suggestions. We greatly appreciate the opportunity to revise and improve our manuscript.

We have carefully considered all comments and recommendations raised during the review process and have revised the manuscript accordingly. In particular, we have corrected textual and terminological inconsistencies, moderated statements that were beyond the evidence generated by the present study, and conducted a comprehensive final cross-check of the numerical values reported throughout the manuscript.

The major revisions made in the present version include:

Additional Editor Comments:

I have considered the revised manuscript together with the reports from Reviewers 2 and 3 and the authors’ point-by-point responses. Both reviewers agree that the major scientific concerns raised during the initial review have been adequately addressed and that the manuscript has improved substantially. I concur with their assessment.

The corrected IL-6 allele and carrier analyses, addition of Hardy-Weinberg equilibrium testing, multivariable logistic regression, and more cautious interpretation of the IL-6, IL-10, and ROC findings have strengthened the manuscript. I have no further major scientific concerns. However, during my final editorial assessment, I identified several minor points that should be addressed to make the manuscript suitable for publication.

1. Please distinguish the crude and adjusted genetic findings more clearly in the Abstract. The reported associations for the IL-6 CC genotype and IL-10 GG genotype are based on unadjusted analyses, whereas neither polymorphism remained independently associated with diabetic neuropathy after multivariable adjustment. This should be stated briefly in the Abstract to provide a balanced summary of the findings.

**Response:** We sincerely thank the Additional Editor for this important observation. We agree that the Abstract should more clearly distinguish the crude genetic associations from the findings of the multivariable analysis. We have therefore revised the Results section of the Abstract to explicitly state that the associations of the IL-6 rs1800796 CC genotype and IL-10 rs1800896 GG genotype with DN were observed in unadjusted analyses, whereas neither polymorphism remained independently associated with DN after multivariable adjustment. We have also clarified that the combined-genotype findings were exploratory. These revisions provide a more balanced summary of the genetic findings and are consistent with the multivariable logistic regression results presented in Table 6.

2. Please confirm whether the continuous variables in Table 2 are presented as mean ± SD or mean ± SEM. Values such as age \(62.64 \pm 7.32\) appear more consistent with standard deviations, although the table identifies them as SEM. The correct measure should be used consistently in the Methods, Results, and tables.

**Response:** We sincerely thank the Additional Editor for identifying this inconsistency. We carefully rechecked the original statistical calculations and confirmed the measures of dispersion used for the continuous variables. We found that the age values in Table 2 had inadvertently been presented as mean ± SD, whereas the remaining continuous variables were presented as mean ± SEM. We have corrected this inconsistency and now report the continuous variables using a single, consistently defined measure throughout the Methods, Results, and tables. The corresponding table headings, footnotes, and Results text have also been revised accordingly. The underlying data and statistical analyses remain unchanged.

3. Please correct the remaining textual and terminological errors. For example, “anthropomorphic” in Table 2 should be “anthropometric,” and “suggesting aa individuals” in the serum IL-6 results should be corrected. Gene, cytokine, genotype, allele, and carrier terminology should also be checked for consistency throughout the manuscript.

**Response:** We sincerely thank the Additional Editor for carefully identifying these remaining textual and terminological inconsistencies. We have thoroughly proofread the manuscript and corrected the identified errors. Specifically, “anthropomorphic” in the title of Table 2 has been corrected to “anthropometric,” and the typographical error “suggesting aa individuals” in the serum IL-6 results has been corrected to “suggesting that individuals...” We also systematically reviewed the terminology used for genes, cytokines, SNPs, genotypes, alleles, and allele carriage throughout the manuscript. The terminology has been standardized to distinguish IL-6/IL-10 as cytokines, IL6/IL10 as genes where appropriate, rs1800796/rs1800896 as SNP identifiers, CC/GC/GG and AA/AG/GG as genotypes, C/G/A as alleles, and allele carriage for presence/absence of specific alleles. Additional grammatical, capitalization, and typographical errors were also corrected throughout the manuscript without altering the underlying data or interpretation.

4. The final paragraph of the Conclusion remains somewhat speculative. Statements that machine learning and precision medicine could “revolutionize” risk assessment or substantially improve clinical outcomes go beyond the evidence presented in this study. Please moderate or remove these claims. The heading “Conclusion and Futuristic Vision” would also be better presented as “Conclusion and Future Directions.”

**Response:** We sincerely thank the Additional Editor for this important observation. We agree that the final portion of the Conclusion was overly speculative relative to the evidence generated by the present study. We have therefore substantially revised the final paragraph to remove statements suggesting that machine learning or precision medicine would “revolutionize” diabetic neuropathy risk assessment or necessarily improve clinical outcomes. These concepts are now presented only as potential areas for future investigation, with emphasis on prospective studies, external validation, and assessment of clinical utility before any potential application. We have also changed the section heading from **“Conclusion and Futuristic Vision”** to **“Conclusion and Future Directions,”** as suggested.

5. Please conduct a final cross-check of all numerical values reported in the Abstract, main text, tables, and figure legends. Table numbering, abbreviations, decimal places, confidence-interval formatting, and p-value presentation should be standardized. The Data Availability Statement should also indicate precisely which underlying numerical data are included in the supplementary material.

**Response:** We sincerely thank the Editor for this careful and important observation. We conducted a final cross-check of all numerical values reported in the Abstract, main text, tables, and figure legends. During this review, we identified and corrected inconsistencies in percentages, odds ratios, confidence intervals, decimal-place presentation, and p-value formatting. We also standardized table and figure numbering, abbreviations, units, confidence-interval formatting, and statistical notation throughout the manuscript. In addition, the Data Availability Statement has been revised to specify precisely which underlying numerical data are provided in the supplementary material.

We have made every effort to address the additional Editor’s concerns thoroughly and to improve the clarity, accuracy, transparency, and scientific presentation of the manuscript. All changes have been incorporated into the revised manuscript for ease of evaluation. We respectfully submit the revised version for further consideration and review. We hope that the revisions satisfactorily address the concerns raised during the review process.

Thank you very much for your time, consideration, and constructive guidance.
